# Dynamic Regulation of the 26S Proteasome: From Synthesis to Degradation

**DOI:** 10.3389/fmolb.2019.00040

**Published:** 2019-06-07

**Authors:** Richard S. Marshall, Richard D. Vierstra

**Affiliations:** Department of Biology, Washington University in St. Louis, St. Louis, MO, United States

**Keywords:** assembly, autophagy, degradation, proteaphagy, proteasome, proteolysis, proteostasis, ubiquitin

## Abstract

All eukaryotes rely on selective proteolysis to control the abundance of key regulatory proteins and maintain a healthy and properly functioning proteome. Most of this turnover is catalyzed by the 26S proteasome, an intricate, multi-subunit proteolytic machine. Proteasomes recognize and degrade proteins first marked with one or more chains of poly-ubiquitin, the addition of which is actuated by hundreds of ligases that individually identify appropriate substrates for ubiquitylation. Subsequent proteasomal digestion is essential and influences a myriad of cellular processes in species as diverse as plants, fungi and humans. Importantly, dysfunction of 26S proteasomes is associated with numerous human pathologies and profoundly impacts crop performance, thus making an understanding of proteasome dynamics critically relevant to almost all facets of human health and nutrition. Given this widespread significance, it is not surprising that sophisticated mechanisms have evolved to tightly regulate 26S proteasome assembly, abundance and activity in response to demand, organismal development and stress. These include controls on transcription and chaperone-mediated assembly, influences on proteasome localization and activity by an assortment of binding proteins and post-translational modifications, and ultimately the removal of excess or damaged particles via autophagy. Intriguingly, the autophagic clearance of damaged 26S proteasomes first involves their modification with ubiquitin, thus connecting ubiquitylation and autophagy as key regulatory events in proteasome quality control. This turnover is also influenced by two distinct biomolecular condensates that coalesce in the cytoplasm, one attracting damaged proteasomes for autophagy, and the other reversibly storing proteasomes during carbon starvation to protect them from autophagic clearance. In this review, we describe the current state of knowledge regarding the dynamic regulation of 26S proteasomes at all stages of their life cycle, illustrating how protein degradation through this proteolytic machine is tightly controlled to ensure optimal growth, development and longevity.

## Roles of the Ubiquitin-Proteasome System and Autophagy in Proteostasis

All cellular organisms require mechanisms to purge unwanted or dysfunctional proteins. In eukaryotes, the autophagy-lysosome and ubiquitin-proteasome systems (UPS) are the two major quality control pathways responsible for maintaining proteome homeostasis and directing recycling to meet nutrient demand. The UPS is typically responsible for degrading short-lived regulatory proteins or soluble mis-folded proteins individually upon insertion into a self-compartmentalized protease, the 26S proteasome (Schubert et al., 2000; Vierstra, 2009; Finley et al., 2012; Samant et al., 2018). By contrast, autophagy can eliminate larger protein complexes, insoluble protein aggregates, and even entire organelles and pathogens *in toto*, due to the sheer size of the engulfing autophagic vesicles (Reggiori and Klionsky, 2013; Gatica et al., 2018; Marshall and Vierstra, 2018a). Substrate selectivity by the UPS is mainly controlled by the attachment of ubiquitin to individual substrates, thus permitting their recognition by ubiquitin-binding proteasome subunits or associated shuttle factors (Finley, 2009; Schreiber and Peter, 2014; Saeki, 2017). For autophagy, equally precise selectively is dictated by a suite of receptors that tether appropriate substrates to the enveloping autophagic membranes (Rogov et al., 2014; Khaminets et al., 2016; Gatica et al., 2018).

Perhaps unsurprisingly given their widespread influence, definitive etiological links exist between various human diseases and mutations in genes that control the UPS and autophagic degradation routes. For example, a decline in both proteasomal and autophagic capacities is associated with aging, neurodegeneration, and other late-onset pathologies, such as Alzheimer's and Parkinson's diseases (Saez and Vilchez, 2014; Dikic and Elazar, 2018; Rape, 2018; Saha et al., 2018; Levine and Kroemer, 2019). On the other hand, the strong dependency of rapidly proliferating cells, such as cancer cells, on active proteasomes has been exploited in therapies that use proteasome inhibitors to differentially induce cell death (Cromm and Crews, 2017; Manasanch and Orlowski, 2017). Similarly, the importance of the UPS and autophagy for efficient nutrient management, seed yield and pathogen defense in crop species underlines its significance to global food security (Vierstra, 2009; Li et al., 2015, 2019; Havé et al., 2017; McLoughlin et al., 2018). As such, knowledge of how the UPS, 26S proteasomes and autophagy are regulated, and of how these systems overlap to ensure proteostasis, is of considerable importance.

## Organization of the Ubiquitin-Proteasome System

**Figure 1:**
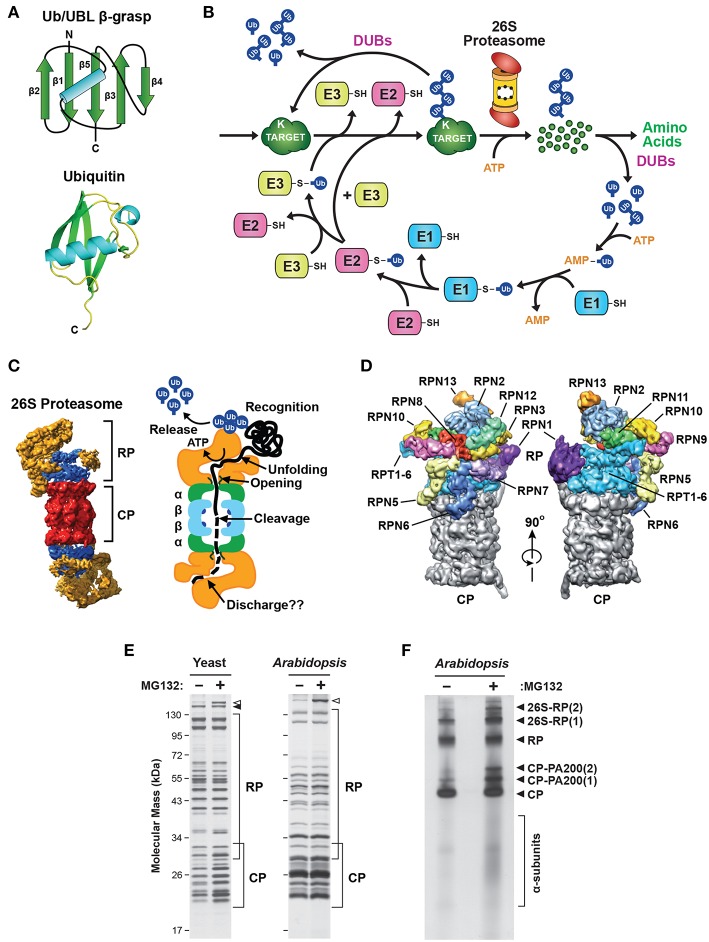
Description of the Ubiquitin-26S Proteasome System (UPS). **(A)** The structure of ubiquitin (Ub). Top, schematic of the β-grasp fold of ubiquitin showing the arrangement of the α-helix and β-strand secondary structures. Bottom, a 3-dimensional ribbon diagram of ubiquitin (Protein Data Bank: 1UBQ). C, carboxyl-terminus. **(B)** Schematic representation of the UPS. The pathway begins with adenosine triphosphate (ATP)-dependent activation of ubiquitin by an E1, followed by transfer of the activated ubiquitin to an E2, and then final attachment of ubiquitin to the target protein with the help of an E3. Typically, the resulting product is a ubiquitin-protein conjugate where the C-terminal glycine carboxyl group of ubiquitin is linked through an isopeptide bond to an accessible ε-amino group of a lysine residue in either the target protein or another ubiquitin molecule. After iterative assembly, the poly-ubiquitylated conjugate can either be disassembled by DUBs, or broken down by the 26S proteasome, in both cases with the concomitant release of the bound ubiquitin moieties intact for re-use. **(C)** 3-dimensional structure of the yeast 26S holo-proteasome, as determined by cryo-EM (Lasker et al., 2012), with the CP shown in red, the RP base shown in blue, and the RP lid shown in yellow (left), and a cartoon representation of the 26S proteasome, highlighting specific functions of the CP and RP during substrate processing (right). **(D)** A detailed view of the subunit architecture of the yeast 26S proteasome RP, as determined by cryo-EM (Lander et al., 2012). The CP is shown in gray, the Rpt ring is shown in light blue, and additional Rpn subunits are shown in various colors with their identity indicated. **(E)** Affinity purification of 26S proteasomes from yeast and *Arabidopsis* showing the size distribution of core subunits. Yeast cells expressing *RPN11-TEV-ProA* (left) or *Arabidopsis* seedlings expressing *PAG1-FLAG* (right) were treated with or without 50 μM MG132 for 16 h before affinity enrichment of 26S proteasomes based on the Protein A or FLAG tags, respectively. The purified particles were then subjected to SDS-PAGE and stained for protein with silver. The distributions of CP and RP subunits are indicated by the brackets. Open and closed arrowheads locate Blm10 and Ecm29, respectively. **(F)** *Arabidopsis* 26S proteasomes affinity-purified as in **(E)** were separated by native gel electrophoresis and stained for protein with silver. The singly- and doubly-capped 26S complex, and the RP, CP, and CP-PA200 sub-complexes, along with partially assembled CP α-subunit rings, are indicated. Images were adapted with permission from Lander et al. (2012), Lasker et al. (2012), Marshall et al. (2015, 2016), and Marshall and Vierstra (2018a).

Ubiquitin is the signature factor within the UPS. It represents the founding member of the β-grasp family of proteins that share a compact, heat-stable domain of ~70 amino acids followed by a protruding C-terminal glycine (Figure 1A). Ubiquitin attachment is achieved through an isopeptide linkage between this glycine and the ε-amino group on the side chain of a surface-exposed lysine residue(s) within the target protein (Ciechanover et al., 1980; Hershko et al., 1980; Wilkinson et al., 1980), although attachment to cysteine, serine or threonine residues, or the N-terminal amino group, have also been reported (Kravtsova-Ivantsiv and Ciechanover, 2012). This conjugation occurs via the sequential actions of three enzyme families that ultimately couple ATP hydrolysis to isopeptide bond formation: the E1 ubiquitin-activating enzymes, the E2 ubiquitin-conjugating enzymes, and the E3 ubiquitin-protein ligases (Figure 1B; Hershko et al., 1983; Vierstra, 2009; Finley et al., 2012). Whereas, the activated E2-ubiquitin intermediate often serves as the immediate donor of ubiquitin, the E3 typically determines which substrate should be ubiquitylated through distinct motifs that separately recognize the substrate and the E2 (Figure 1B).

To date, four main types of E3 have been described, classified by their mechanism(s) of action and subunit composition: HECT, RING, U-box, and RING-between-RING (RBR). The RING family of E3s includes the multi-subunit Cullin-RING ligases (CRLs) that exploit one of several Cullin isoforms to scaffold the complex. Importantly, eukaryotes have evolved hundreds or even thousands of distinct E3s bearing a wide variety of substrate-recognition elements connected to a small number of common scaffolds (Hua and Vierstra, 2011; Buetow and Huang, 2016; Zheng and Shabek, 2017). This remarkable diversity allows individual E3s to operate in distinct cellular contexts, respond to unique cellular signals, and process a diverse array of protein substrates.

The final products of this conjugation cascade can be proteins modified with a single ubiquitin (mono-ubiquitylation), with several single ubiquitin moieties (multi-ubiquitylation), and/or with chain(s) of ubiquitin that are covalently concatenated via any of seven internal lysines or the N-terminus (poly-ubiquitylation; Kirisako et al., 2006; Xu et al., 2009; Yau et al., 2017). Such complexity allows for a myriad of functions triggered by ubiquitylation, including some that are not connected to proteolysis, through the use of distinct classes of receptors that recognize specific ubiquitin chain topologies (Husnjak and Dikic, 2012; Lu et al., 2015; Oh et al., 2018). The UPS also includes a diverse collection of deubiquitylating enzymes (DUBs) specific for various types of ubiquitin linkages and/or substrates. These DUBs uniquely release both the target and the ubiquitin moieties intact (Figure 1B), thus allowing ubiquitylation to function in a reversible manner (de Poot et al., 2017; Clague et al., 2019). However, in most cases ubiquitylated substrates are recognized via their attached ubiquitin(s) and degraded by the 26S proteasome, an ATP-dependent proteolytic machine that cleaves the substrate into short peptides concomitant with release of the ubiquitin moieties by associated DUBs for re-use (Figures 1B,C). Here, proteins modified with poly-ubiquitin chains internally linked through K11 or K48 appear to be the favored substrates (Yau et al., 2017; Samant et al., 2018). As will be described below, our emerging appreciation of this proteolytic complex has revealed how it also contributes to the regulation and specificity of the UPS beyond E3s.

## Composition of the 26S Proteasome

At the heart of the UPS is the 26S proteasome, a 2.5 MDa, multi-subunit protease located in the cytosol and nucleus of all eukaryotic cells (Reits et al., 1997; Enenkel et al., 1998; Russell et al., 1999; Brooks et al., 2000; Pack et al., 2014; Marshall et al., 2015). The exceptional complexity and size of this proteolytic machine have made it an excellent model for understanding how intricate macromolecular structures are co-ordinately assembled rapidly and faithfully from dozens of components. Much of our current understanding of proteasome architecture has arisen from exceptionally well-resolved 3-dimensional models that have continually improved in step with rapid advances in X-ray crystallographic and cryo-electron microscopic (EM) imaging (Baumeister et al., 1994; Groll et al., 1997; Nickell et al., 2009; Lander et al., 2012; Lasker et al., 2012; de la Peña et al., 2018; Dong et al., 2019).

26S proteasomes are composed of two functionally distinct sub-complexes that are separately stable (Figures 1C,D); the 20S core protease (CP) that houses the peptidase activities, capped at one or both ends by the 19S regulatory particle (RP) that captures and prepares appropriate substrates for breakdown (Groll et al., 1997; Finley, 2009; Book et al., 2010; Lander et al., 2012; Lasker et al., 2012; Bhattacharyya et al., 2014; Collins and Goldberg, 2017; Rousseau and Bertolotti, 2018; Finley and Prado, 2019). The CP has a barrel shape generated by four stacked hetero-heptameric rings, which contain seven α-subunits or seven β-subunits in a C2 symmetric α_1−7_/β_1−7_/β_1−7_/α_1−7_ configuration (Figure 1C). Upon assembly, a central chamber is formed at the interface of the β-rings that houses six catalytic sites responsible for peptide bond cleavage, provided by the β_1_, β_2_ and β_5_ subunits (Arendt and Hochstrasser, 1997; Heinemeyer et al., 1997; Dick et al., 1998; Kisselev et al., 1999, 2003). These active sites consist of a novel catalytic triad formed by an N-terminal threonine that becomes exposed during CP assembly by proteolytic removal of a proximal propeptide (Chen and Hochstrasser, 1996; Schmidtke et al., 1996; Seemuller et al., 1996; Huber et al., 2016; Li et al., 2016). Collectively, these CP peptidases can cleave a broad array of polypeptides, with the β_1_, β_2_ and β_5_ active sites providing trypsin-like, chymotrypsin-like and caspase-like cleavage properties, respectively (Arendt and Hochstrasser, 1997; Heinemeyer et al., 1997; Nussbaum et al., 1998; Groll et al., 1999; Kisselev et al., 1999.)

Additionally, more specialized β-subunits have been identified in mammalian cells that are expressed and incorporated into the CP to confer slightly altered catalytic preferences to proteasomes (Murata et al., 2018). The thymo-proteasome is found only in cortical epithelial cells of the thymus, and is thought to play a vital role in the positive selection of CD8^+^ T-cells through lower chymotrypsin-like activity from the β_2_ subunit (Murata et al., 2007). Immuno-proteasomes are enriched in a variety of immune system-related tissues, such as the spleen, thymus, lung, liver, kidney, colon, small intestine and antigen-presenting cells. Their expression can also be induced in non-immune tissues in response to specific stimuli, such as interferon-γ (Gaczynska et al., 1993; Hisamatsu et al., 1996). Immuno-proteasomes preferentially cleave after basic and hydrophobic residues through replacement of the β_1_, β_2_ and β_5_ subunits with closely-related isoforms (known as LMP2, MECL1 and LMP7/PSMB11, respectively), leading to release of peptides more favorable to MHC class I antigen-presenting receptors (Driscoll et al., 1993; Kincaid et al., 2011; Huber et al., 2012). Whether other eukaryotes besides mammals exploit β-subunit diversity to alter proteasome activity and function is not yet known.

On top of the β-subunit rings sit the α-subunit rings (Figure 1C), which create two antechambers with narrow opposing axial pores that are gated by extensions at the N-terminus of several α-subunits (Groll et al., 2000; Köhler et al., 2001; Smith et al., 2005; da Fonseca and Morris, 2008; Rabl et al., 2008; Ruschak et al., 2010). Occlusion of the pores is mainly attributed to the N-terminal extension of the α_3_ subunit, since its deletion creates a constitutively open pore (Groll et al., 2000). Gate opening in the holo-proteasome is normally triggered by docking of the CP to various proteasome regulators, such as the multi-subunit RP (PA700), or the activators PA28αβ, PA28γ, PA200 (also known as Blm10), PI31 (also known as PSMF1, Fub1 or PTRE1), and Cdc48 (also known as VCP or p97; Dubiel et al., 1992; Zaiss et al., 1999; Li and Rechsteiner, 2001; Schmidt et al., 2005; Barthelme and Sauer, 2012; Esaki et al., 2018). These regulators (or one or more of their subunits) typically possess a C-terminal HbYX motif (where Hb represents a hydrophobic residue, Y is tyrosine, and X is any amino acid) that inserts into pockets formed at the interfaces between adjacent α-subunits (Smith et al., 2005, 2007; Rabl et al., 2008; Sadre-Bazzaz et al., 2010; Tian et al., 2011; Park et al., 2013). Through this distinctive and stable architecture, the CP acts as a self-compartmentalized protease that only degrades polypeptides that are deliberately recognized, unfolded, and imported into the β-ring chamber.

The main CP regulator is the RP, which loosely binds to either or both ends of the CP in the presence of ATP (Eytan et al., 1989; Armon et al., 1990; Smith et al., 2005; Liu et al., 2006). The RP sits over the axial pores of the CP and provides activities for recognizing ubiquitylated substrates, driving their unfolding, opening the α-ring pore, importing substrates into the CP, and finally releasing the ubiquitin moieties prior to substrate degradation (Figures 1C,D; Bhattacharyya et al., 2014; Collins and Goldberg, 2017; Finley and Prado, 2019).

The RP can be separated into two sub-complexes *in vitro*, termed the base and the lid. The base directly contacts the CP and contains a ring of AAA-ATPases (Rpt1-6) plus four non-ATPase subunits, Rpn1, Rpn2, Rpn10 and Rpn13, while the more peripheral lid is composed of an additional 10 non-ATPase subunits [Rpn3, 5, 6, 7, 8, 9, 11, 12, and Sem1 (also known as Rpn15/Dss1)] with varying functions (Figures 1C,D; Glickman et al., 1998; Finley, 2009; Book et al., 2010; Lander et al., 2012; Lasker et al., 2012; Bhattacharyya et al., 2014). Association of the Rpt ring with the heptameric α-ring is slightly out of register given the unequal number of subunits, which leads to a loose and tilted contact that might help with substrate processing (Smith et al., 2011; Tian et al., 2011). Engaging substrates both enforces the CP and RP association, leading to the enrichment of singly- and double-capped 26S proteasomes, and appears to alter the CP-RP contact (Chen et al., 2016; Wehmer et al., 2017; Eisele et al., 2018). These features provide a visual method to assess whether 26S proteasomes are actually engaged with substrates *in vivo*. When applied to neuronal cells under non-stressed conditions, it was found that only ~20% of the particles were actively processing substrates, suggesting that the capacity of 26S proteasomes is often under-utilized (Asano et al., 2015).

The lid-base demarcation of the RP was first revealed by the absence of lid subunits in proteasomes isolated from Δ*rpn10* yeast cells, and hence it was thought that Rpn10 helps maintain the lid-base contact (Glickman et al., 1998). However, more recent structural studies showed that Rpn10 has an indirect stabilizing effect within the RP by binding Rpn9. The lid-base association is instead mainly enforced by the Rpn3, Rpn7, Rpn8, and Rpn11 cluster (Lander et al., 2012; Lasker et al., 2012; Bhattacharyya et al., 2014). Besides the HbYX motifs in the Rpt ring, Rpn6 provides a molecular clamp to anchor the RP onto the CP (Pathare et al., 2012).

The ring of Rpt subunits couples ATP hydrolysis to substrate unfolding (de la Peña et al., 2018; Eisele et al., 2018; Dong et al., 2019), and repositions the extensions of the CP α-subunits to permit entry through the axial pore (Smith et al., 2005, 2007; Rabl et al., 2008; Tian et al., 2011). The coiled-coil regions of adjacent Rpt pairs also intertwine to create three spokes onto which most Rpn subunits are scaffolded (Lander et al., 2012; Lasker et al., 2012). A key RP subunit is Rpn11, a DUB that uniquely employs a zinc-containing active site to catalyze the release of poly-ubiquitin chains isopeptide-linked to substrates (Verma et al., 2002; Yao and Cohen, 2002; Pathare et al., 2014; Worden et al., 2014, 2017). Through Rpn11 and other loosely associated DUBs, such as Ubp6/USP14 and UCH37/UCHL5 (Leggett et al., 2002; Hanna et al., 2006; Aufderheide et al., 2015a; Bashore et al., 2015; Lee et al., 2016; de Poot et al., 2017), bound ubiquitin moieties are actively released for re-use before substrate hydrolysis, thus helping to promote substrate degradation by preventing the unusually stable structure of ubiquitin from impeding translocation into the CP (Verma et al., 2002; Yao and Cohen, 2002; Worden et al., 2017).

Another intriguing CP regulator is the evolutionarily conserved protein known as PI31/PSMF1 in mammals (Chu-Ping et al., 1992; Zaiss et al., 1999; McCutchen-Maloney et al., 2000), Fub1 in yeast (Hatanaka et al., 2011; Yashiroda et al., 2015), and PTRE1 in plants (Yang et al., 2016), which for the animal form uses multiple structural features, including a HbYX motif, to bind the CP α-ring (Li et al., 2014). It was originally described as a negative regulator of the proteasome, based on its ability to suppress CP activity *in vitro* (Chu-Ping et al., 1992; McCutchen-Maloney et al., 2000; Li et al., 2014; Yashiroda et al., 2015). However, it is now considered to have little effect on proteasome activity *in vivo* (Li et al., 2014; Yashiroda et al., 2015), and may even be an activator of the 26S holo-proteasome under certain conditions (Bader et al., 2011; Cho-Park and Steller, 2013; Yang et al., 2016). Interestingly, ADP-ribosylation of *Drosophila melanogaster* PI31 by the ADP-ribosyltransferase tankyrase was shown to promote 26S proteasome activity by both reducing the affinity of PI31 for CP α-subunits, thus permitting CP-RP association, and by increasing the affinity of PI31 for the RP assembly chaperones Nas2 and Hsm3 (Cho-Park and Steller, 2013). However, no evidence was found to support a role for ADP-ribosylation in mammalian PI31 function (Li et al., 2014). In *Arabidopsis*, PTRE1 is an abundant co-factor of 26S proteasomes, and its deletion generates an auxin hyposensitive phenotype, with elevated levels of the AUX/IAA family of auxin-response repressors and reduced activity of the 26S proteasome, suggesting that it promotes signaling from this central plant hormone by controlling UPS-mediated AUX/IAA protein turnover (Yang et al., 2016).

Our understanding of 26S proteasome composition in a variety of species has been greatly aided by the use of tagged subunits that allow rapid affinity purification of the complex (Figure 1E; Leggett et al., 2002, 2005; Book et al., 2010; Marshall et al., 2017). Proteomic analysis of the resulting preparations not only identified the core α, β, Rpt and Rpn subunits, but also a large collection of regulators and assembly chaperones (Leggett et al., 2002; Book et al., 2010). Furthermore, by conducting purifications in the absence of ATP, it is relatively easy to obtain preparations enriched in either the CP or RP sub-complexes (Leggett et al., 2005). Singly- and doubly-capped 26S particles, plus the CP, RP and Blm10/PA200-CP sub-complexes, can also be visualized following native PAGE (Figure 1F), with proteolytically active complexes then identified *in situ* with fluorogenic peptide substrates (Elsasser et al., 2005).

## Recognition of Ubiquitylated Substrates by the 26S Proteasome

An important aspect of proteasomal degradation involves controlling access of substrates to the CP proteolytic chamber. Substrate selection is dictated by several ubiquitin receptors intrinsic to the RP, including Rpn1, Rpn10, Rpn13, and possibly Sem1 (van Nocker et al., 1996a,b; Verma et al., 2004; Finley, 2009; Fatimababy et al., 2010; Sakata et al., 2012; Paraskevopoulos et al., 2014; Shi et al., 2016; Saeki, 2017). Rpn10 recognizes ubiquitin via a defined ubiquitin-interacting motif (UIM; Haracska and Udvardy, 1997; Fu et al., 1998b; Hofmann and Falquet, 2001; Verma et al., 2004), and is unique among proteasome subunits in that it exists as both proteasome-bound and free forms (van Nocker et al., 1996a,b; Haracska and Udvardy, 1997; Marshall et al., 2015). Rpn13 recognizes ubiquitin via an N-terminal pleckstrin-like receptor for ubiquitin (PRU) domain, which is structurally distinct from UIMs but binds to the same hydrophobic patch on ubiquitin (Husnjak et al., 2008; Schreiner et al., 2008). The C-terminal domain of human RPN13 binds to and activates the DUB UCH37 (Hamazaki et al., 2006; Yao et al., 2006), and together they provide a “proof-reading” activity that permits escape of poorly or inadvertently ubiquitylated substrates through release of the bound ubiquitin moieties. More recently, Rpn1 and Sem1 were reported to be proteasomal ubiquitin receptors (Paraskevopoulos et al., 2014; Shi et al., 2016; Dong et al., 2019). However, it remains unclear whether Sem1 can recruit ubiquitylated proteins to the 26S proteasome, because the purported ubiquitin-binding surface in this intrinsically disordered protein overlaps with its proteasome-binding surface (Shi et al., 2016).

In addition to these core ubiquitin receptors, there are several extra-proteasomal ubiquitin-binding proteins that shuttle ubiquitylated cargo to the RP. These work by virtue of one or more C-terminal ubiquitin-associated (UBA) domains that bind ubiquitin (Hofmann and Bucher, 1996; Wilkinson et al., 2001), coupled to an N-terminal ubiquitin-like (UBL) domain that binds to the ubiquitin receptors within the proteasome (Schauber et al., 1998; Elsasser et al., 2002, 2004; Walters et al., 2002; Husnjak et al., 2008; Chen et al., 2019). Because the UBL and UBA domains are typically joined through a long, flexible linker region, it is thought that these shuttle factors allow greater orientational freedom of proteasome-bound substrates as compared to direct docking.

Important UBL-UBA shuttle factors include Rad23, Dsk2 and Ddi1, which are conserved in plants, fungi and mammals (Finley, 2009; Fatimababy et al., 2010; Hjerpe et al., 2016; Saeki, 2017; Samant et al., 2018). Indeed, a recent proteomics study concluded that the UBL-UBA shuttle factors are the major route by which proteins are targeted to the proteasome in yeast (Tsuchiya et al., 2017). Even though UBL-UBA proteins interact with 26S proteasomes, they are immune to degradation, which at least for Rad23 appears to require its C-terminal UBA domain (Heessen et al., 2005; Heinen et al., 2011) and the absence of an unstructured region for initiating degradation (Fishbain et al., 2011). Interestingly, yeast strains in which the *RAD23, DSK2* and *DDI1* genes have been deleted, and the ubiquitin-binding elements of Rpn1, Rpn10 and Rpn13 have been removed by mutation, are sensitive to stress but are nevertheless viable and still capable of degrading ubiquitylated substrates, suggesting that additional ubiquitin receptors for the proteasome remain to be identified (Shi et al., 2016).

Substrate breakdown by proteasomes is further regulated by various post-translational modifications impacting the layers of intrinsic and extrinsic ubiquitin receptors. For example, in response to proteasome inhibition or conditions that impair proteasome function, human RPN13 becomes ubiquitylated by the proteasome-associated E3 UBE3C, which prevents substrate binding (Besche et al., 2014). Mono-ubiquitylation of Rpn10 likewise dampens its ability to bind ubiquitylated substrates and shuttle factors (Isasa et al., 2010; Lipinszki et al., 2012; Jacobson et al., 2014; Zuin et al., 2015), while the interaction of Rad23 with the proteasome is inhibited by phosphorylation of its UBL domain (Liang et al., 2014), thus controlling how effectively 26S proteasomes can capture their targets.

Although it was long believed that ubiquitylation is sufficient to mark a protein for degradation, it is now accepted that turnover also requires elements within both the 26S proteasome and the substrate, most notably an unstructured region near the end of the polypeptide awaiting breakdown that is recognized by features within the RP base (Lee et al., 2001; Prakash et al., 2004; Yu et al., 2016). In particular, the ability of 26S proteasomes to recognize both a poly-ubiquitin chain and an unstructured region likely provides the basis for determining which proteins should be degraded and which to spare. This critical decision requires two steps; an initial step in which the attached poly-ubiquitin chain undergoes reversible binding to ubiquitin receptors associated with the RP, followed by a second step where the ubiquitylated substrate binds more tightly depending on accessibility of the unstructured region to the Rpt ring (Peth et al., 2010; Collins and Goldberg, 2017). This reaction sequence provides an opportunity for competing processes to determine the fate of the substrate. For example, multiple DUBs can promote the release of some, perhaps many, ubiquitylated proteins that initially bind only weakly (Lee et al., 2016). Conversely, if a substrate becomes tightly bound through its unstructured region, the unfoldase activity of the AAA-ATPase ring of the RP is engaged (Peth et al., 2013a), and the partially unfolded substrate is then locked into the route leading to its destruction. Clearly, not all proteins contain an unstructured region capable of initiating degradation; in these cases, the AAA-ATPase activity of Cdc48/p97 is thought to assist in unraveling well-folded proteins as a prelude to breakdown (Olszewski et al., 2019).

Substrate degradation ultimately requires release of the bound ubiquitin, which provides an additional control step (de Poot et al., 2017). Deubiquitylation is performed by proteasome-associated DUBs, including one DUB intrinsic to the proteasome (Rpn11), and others that transiently associate (Ubp6/USP14 and UCH37/UCHL5). Rpn11 releases the poly-ubiquitin chain intact after the substrate irreversibly engages with the proteasome entry channel (Verma et al., 2002; Yao and Cohen, 2002; Pathare et al., 2014; Worden et al., 2014, 2017). The other DUBs favor progressive trimming of ubiquitin chains, with the balance between ubiquitin removal and ubiquitin addition by proteasome-interacting E3s such as yeast Hul5 dictating either substrate degradation or release (Leggett et al., 2002; Crosas et al., 2006; Bashore et al., 2015; Lee et al., 2016).

## Transcriptional Regulation of 26S Proteasome Subunit Abundance

**Figure 2:**
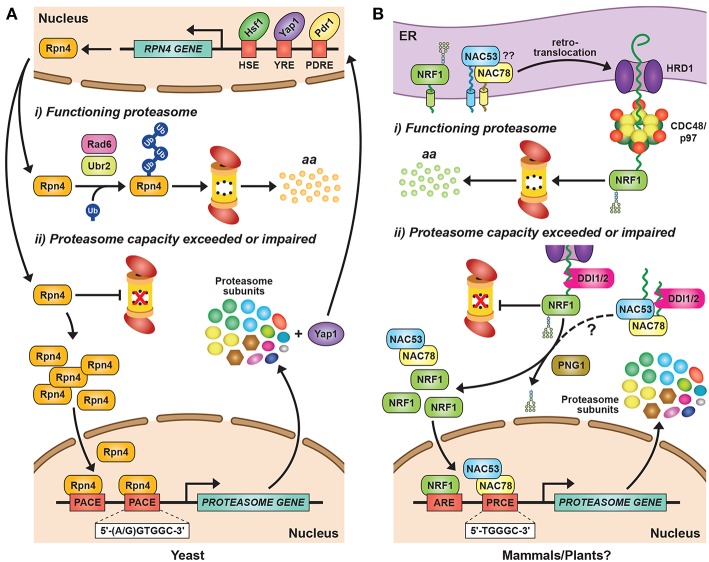
Transcriptional Regulation of Proteasome Subunit Genes. **(A)** Regulation of proteasome gene expression in yeast. Expression of the transcription factor Rpn4 is controlled by various *cis*-regulatory elements bound by transcription factors, such as Hsf1, Pdr1, and Yap1. Rpn4 has an extremely short half-life and is continuously ubiquitylated and degraded by the 26S proteasome under normal growth conditions, a pathway initiated by the E2 Rad6 and the E3 Ubr2. A ubiquitin-independent route for Rpn4 degradation also exists. When proteasome capacity is exceeded or impaired, Rpn4 is stabilized and translocates to the nucleus, where it binds to a hexameric consensus nucleotide sequence [(A/G)GTGGC], known as the proteasome-associated control element (PACE), present in the promoters of most proteasome subunit genes. This binding leads to increased expression of proteasome subunits, along with additional genes involved in protein ubiquitylation, DNA repair, and other stress responses. One of the latter genes encodes Yap1, which can further increase Rpn4 levels via a positive-feedback loop. HSE, heat shock element; YRE, Yap response element; PDRE, pleiotropic drug response element; aa, amino acids. **(B)** Regulation of proteasome gene expression in mammals and plants. The mammalian transcription factor NRF1 is a type II endoplasmic reticulum (ER) membrane protein that is continuously retro-translocated to the cytosol via the ER-associated protein degradation (ERAD) pathway, a process requiring activity of the E3 ligase HRD1 and the AAA-ATPase CDC48/p97. Retro-translocated NRF1 is rapidly ubiquitylated and degraded by the 26S proteasome. When proteasome capacity is exceeded or impaired, NRF1 is stabilized during retro-translocation, where it is cleaved by the aspartyl protease DDI2. The resulting active form of NRF1 is deglycosylated by PNG1 and then translocates to the nucleus, where it binds antioxidant response elements (AREs) to activate transcription of its target genes, including those encoding proteasome subunits. The *Arabidopsis* transcription factors NAC53 and NAC78 control proteasome subunit gene expression and are predicted to be ER-localized transmembrane proteins. Given that *Arabidopsis* DDI1 also contains an aspartyl protease domain, we predict that transcriptional regulation of the proteasome in plants proceeds by a similar mechanism as in mammalian cells, by which the processed NAC53/78 dimer enters the nucleus and binds to proteasome-related *cis*-elements (PRCEs) to activate transcription.

Synthesis of 26S proteasomes is energetically costly given their complexity and abundance and, as a consequence, cells have evolved sophisticated mechanisms to ensure an adequate supply of functioning particles. In fact, proteasomes comprise as much as 1% of total protein in certain mammalian cell types (Tanaka and Ichihara, 1989). The main control point is through regulated expression of the corresponding suite of proteasome subunits and associated genes, which is tightly co-ordinated in an attempt to provide stoichiometric amounts of each polypeptide (Figure 2). How tight this regulation is within the collection of proteasome genes remains unclear, as excess subunits do not typically accumulate within cells as free forms (the exception being Rpn10), and appear to be rapidly degraded if they fail to integrate into their respective CP or RP sub-complexes (Peters et al., 2015; Nahar et al., 2019). Thus, while transcription and translation are modulated in an attempt to provide stoichiometric expression, an important arbiter dictating the final concentration of proteasomes might be the abundance of one or more factors in limiting supply. Nevertheless, multiple studies have documented the concerted transcriptional regulation of proteasome genes during development or in response to stress, and have contributed to a growing body of evidence for common signaling pathways regulating their expression.

In yeast, mammals and plants, the controlled expression of proteasome subunit genes is achieved by the unrelated but functionally analogous transcription factors Rpn4, NRF1/2, and NAC53/78, respectively (Figure 2). This regulation is best understood in yeast, where the C2H2-type zinc finger transcription factor Rpn4 binds to a six nucleotide sequence [(A/G)GTGGC)] known as the proteasome-associated control element (PACE) that can be found in the promoter region of genes encoding most proteasome subunits and related factors (Mannhaupt et al., 1999; Xie and Varshavsky, 2001; Shirozu et al., 2015). Rpn4 has an extremely short half-life due to rapid proteasomal degradation (Xie and Varshavsky, 2001). However, when proteasome capacity fails to keep up with demand, Rpn4 turnover slows, leading to a rise in its levels and a concomitant increase in proteasome gene expression (Figure 2A). Rpn4 itself is integrated into a broader stress-responsive regulatory network, including controls on *RPN4* gene expression by several transcription factors including Hsf1, Pdr1, Pdr3, and Yap1 (Figure 2A; Owsianik et al., 2002; Hahn et al., 2006).

The proteasomal degradation of Rpn4 under low proteasome demand is mediated by two distinct degrons, both of which must be blocked to stabilize Rpn4 (Ju and Xie, 2004). One degron is independent of ubiquitin (Ha et al., 2012), while the second relies on phosphorylation-induced ubiquitylation of specific lysines via the E2 Rad6 and the E3 Ubr2 (Wang et al., 2004; Ju and Xie, 2006; Ju et al., 2007). The ubiquitin-independence of one breakdown route is unusual for a short-lived protein, but it might ensure that Rpn4 is sensitive principally to fluctuations in proteasome activity, rather than ubiquitin availability, which is separately regulated (Hanna et al., 2007). Controlling Rpn4 levels and activity, and hence proteasome abundance, is critical for yeast survival in response to multiple stresses, including DNA damage, proteotoxic stress, and changes in redox balance (Wang et al., 2008, 2010a; Ma and Liu, 2010).

A similar regulatory loop exists in mammalian cells, where a concerted increase in the expression of proteasome subunits is observed in response to proteasome inhibition (Meiners et al., 2003). However, the lack of obvious mammalian orthologs of Rpn4 and PACE sequences within proteasome subunit genes suggested early on that novel mechanism(s) are in play. It is now clear that the transcription factors NF-Y, FOXO4 and STAT3 collectively drive the constitutive expression of proteasome genes (Vilchez et al., 2012; Xu et al., 2012; Vangala et al., 2014). NF-Y dictates the expression of loci encoding six CP subunit (α_2_, α_5_, α_7_, β_3_, β_4_ and β_6_), five RP subunits (RPT1, RPT5, RPT6, RPN10, and RPN11), and one assembly chaperone (NAS6/p28), each of which contains one or more CCAAT *cis*-elements in their promoter regions (Xu et al., 2012). FOXO4 promotes RPN6 expression, which contributes to high proteasome activity in pluripotent stem cells (Vilchez et al., 2012), while STAT3 regulates the expression of numerous β-subunit genes (Vangala et al., 2014).

Additionally, two basic leucine zipper family transcription factors appear to fulfill the role of yeast Rpn4 in up-regulating proteasome gene expression when capacity is impaired: nuclear factor erythroid 2-related factor 1 (NRF1, also known as NFE2L1) and, to a lesser extent, NRF2 (Radhakrishnan et al., 2010; Steffen et al., 2010; Lee C. S. et al., 2011; Koizumi et al., 2018). Chromatin immunoprecipitation (ChIP)-seq experiments identified (A/G)TGACTCAGC as the consensus binding site for NRF1 in mice (Baird et al., 2017), which notably exists in the enhancer or promoter regions of all proteasome subunit genes.

Similar to yeast Rpn4, NRF1 is rapidly degraded by the UPS, albeit via a different mechanism (Figure 2B). NRF1 is a type II integral ER membrane protein (Wang and Chan, 2006; Zhang et al., 2007) that is retro-translocated continuously from the ER back to the cytosol under normal conditions via the ER-associated protein degradation (ERAD) pathway, where it is rapidly ubiquitylated and removed by 26S proteasomes (Figure 2B; Steffen et al., 2010; Radhakrishnan et al., 2014; Sha and Goldberg, 2014). This turnover requires ubiquitylation of NRF1 by the ER-resident E3 HRD1 [which also acts as the retro-translocation channel (Schoebel et al., 2017)], and subsequent extraction by Ccd48/p97 (Steffen et al., 2010; Radhakrishnan et al., 2014). When proteasomal capacity is limited, NRF1 stalls during retro-translocation and is instead deglycosylated and proteolytically liberated from the ER in an active form that subsequently translocates into the nucleus to drive transcription (Figure 2B; Radhakrishnan et al., 2014; Sha and Goldberg, 2014; Lehrbach et al., 2019).

After some initial controversy regarding the identity of the responsible protease (Sha and Goldberg, 2014, 2016; Vangala et al., 2016), it is now clear that this cleavage is performed by the UBL-UBA protein DDI2, using the aspartyl protease activity provided by its distinctive retroviral protease-like domain (Figure 2B; Koizumi et al., 2016). A likely scenario is that this shuttle factor selectively recognizes ubiquitylated NRF1 through their ubiquitin-binding capacities and then direct its cleavage. An analogous mechanism exists in *Caenorhabditis elegans* (Lehrbach and Ruvkun, 2016, 2019), suggesting that this activation mechanism is widely conserved among animals. Once inside the nucleus, NRF1 stability is additionally regulated by at least two CRL E3s that trigger its ubiquitylation and subsequent degradation, with this turnover also sensitive to proteasome capacity (Biswas et al., 2011; Tsuchiya et al., 2011).

In *Arabidopsis*, the co-ordinated expression of proteasome subunit genes is controlled by at least two transcription factors from the NAM/ATAF1/CUC2 (NAC) family, NAC53 and NAC78 (Yabuta et al., 2011; Nguyen et al., 2013; Gladman et al., 2016). NAC78 (also known as NTL11 or RPX1) was initially identified as a gene whose expression was up-regulated in response to intense light and heat stress (Nishizawa et al., 2006; Morishita et al., 2009), and whose knock-out increased leaf organ size (Nguyen et al., 2013). A role in proteasome gene expression was then identified by over-expression studies showing that NAC78 positively regulates the expression of core proteasome subunit genes, and that its putative DNA-binding site [TGGGC, known as the proteasome-related *cis*-element (PRCE)] is present within many, but not all, associated promoters (Morishita et al., 2009; Yabuta et al., 2011; Nguyen et al., 2013). Interestingly, while many proteasome subunits are encoded by paralogous genes in *Arabidopsi*s and other plants (Fu et al., 1998a, 1999; Shibahara et al., 2002; Yang et al., 2004; Book et al., 2010), often only one gene of a pair is responsive to NAC78 over-expression or treatment with proteasome inhibitors (Gladman et al., 2016), suggestive of non-redundancy. Besides proteasome genes, an extended collection of genes encoding proteasome accessory proteins, assembly chaperones, autophagy components, and detoxifying enzymes are also included within the “proteasome stress” regulon, suggesting that plant cells use an assortment of strategies to combat proteasome insufficiency besides assembling more particles (Gladman et al., 2016).

Promoter-binding and phylogenetic analyses identified a close homolog of NAC78, termed NAC53 (also known as NTL4) that works in concert (Gladman et al., 2016). The two proteins interact, and the elimination of both, but not each individually, severely impairs up-regulation of the proteasome stress regulon in response to proteasome inhibition, rendering the double *nac53 nac78* mutant plants hyper-sensitive to CP inhibitors such as MG132 and bortezomib (Figure 2B). Given that NAC53 and NAC78 are predicted to possess a C-terminal transmembrane domain, and that other members of the membrane-bound NAC family have been reported to use proteolytic release from membrane stores to regulate their transcriptional activity (Kim et al., 2007), we predict that a cleavage mechanism similar to that employed to release mammalian NRF1 from membranes operates in plants (Figure 2B). In support, *Arabidopsis* harbors a homolog of DDI2 (Farmer et al., 2010) that could use its internal retroviral protease domain to cleave NAC53 and NAC78, thus permitting their release from the ER and entry into the nucleus where they would then activate the proteasome stress regulon (Figure 2B).

## Regulated Assembly of the Proteasome Core Protease

**Figure 3:**
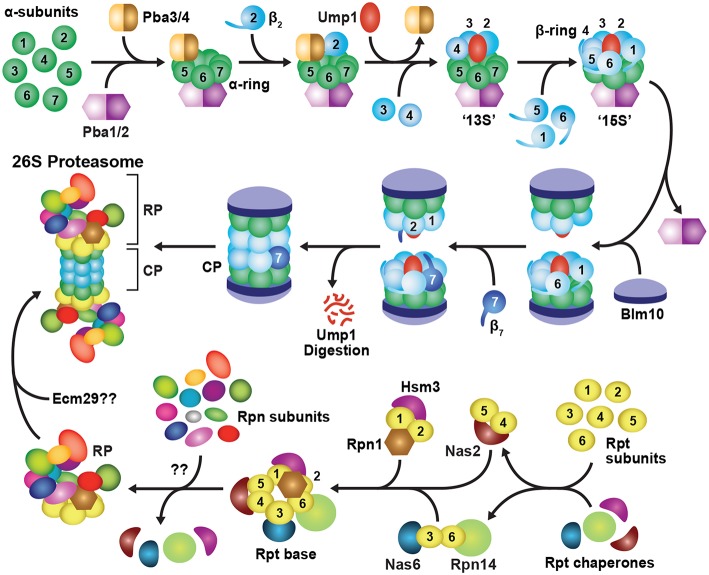
Assembly Pathway for 26S Proteasomes in Yeast. Formation of the CP begins with assembly of the α-subunit ring, which is mediated by two hetero-dimeric chaperone complexes, Pba1-Pba2 and Pba3-Pba4. Upon α-ring completion, the CP β-subunits are incorporated in a specific order, starting with β_2_ followed by β_3_, β_4_, β_5_, β_6_ and β_1_, resulting in sequential formation of 13S, 15S and half-proteasome intermediates. Assembly of the β-ring is assisted by the Ump1 chaperone, and the resulting half-proteasome is capped by Blm10. The β_7_ subunit is then incorporated, which promotes the association of two half-proteasomes to generate a complete CP. Auto-catalytic removal of the β-subunit propeptides then activates the CP and leads to Ump1 degradation. The RP base assembles from three separate chaperone modules, namely Nas2-Rpt4-Rpt5, Nas6-Rpt3-Rpt6-Rpn14, and Hsm3-Rpt1-Rpt2-Rpn1. These modules associate with one another in an ordered manner to construct the Rpt ring, followed by incorporation of Rpn2, Rpn13, and finally Rpn10 to form the completed base. The RP lid assembles largely spontaneously, beginning with dimerization of Rpn8 and Rpn11, followed by sequential recruitment of Rpn6, Rpn5, Rpn9, a trimeric Rpn3-Rpn7-Sem1 complex, and finally, Rpn12. The lid and base then combine to form a complete RP. Upon completion of CP and RP assembly, the two sub-complexes associate to form the mature 26S holo-proteasome. This association occurs via insertion of C-terminal HbYX motifs from the Rpt subunits into pockets between adjacent CP α-subunits. Finally, correct CP-RP association is confirmed by an Ecm29-mediated checkpoint.

Assembly of the holo-26S proteasome following subunit synthesis is a highly complex process that requires numerous dedicated chaperones and maturation factors (Figure 3; Howell et al., 2017; Rousseau and Bertolotti, 2018). Construction of the CP and the Rpt ring of the RP are particularly challenging as compared to their bacterial and archeal counterparts, due to diversification of the α, β and Rpt subunits. This heterogeneity imposes positional constraints on the ordered assembly of the corresponding α and β heptameric rings and the Rpt hexameric ring, and subsequent docking of these rings in correct register with each other. As such, proteasome assembly is a relatively slow process, with an experimentally determined half-time of around 20 min in yeast (Chen and Hochstrasser, 1996), and between 30 and 80 min in mammalian cells (Yang et al., 1995; Heink et al., 2005; Hirano et al., 2005). Because the individual subunits of the α, β and Rpt rings share substantial sequence and structural similarity, having likely evolved from a common ancestor (Gille et al., 2003), mis-assembly can and does occur, leading to faulty assembly intermediates that sterically occlude or otherwise interfere with construction and/or activity of the CP and/or RP (Gerards et al., 1997, 1998; Yao et al., 1999; Takeuchi and Tamura, 2004; Ishii et al., 2015). Thus, mechanisms to limit the formation of these dysfunctional products, and remove any that arise inadvertently, are essential for maintaining a healthy proteasome pool.

CP assembly begins with formation of individual α-rings (Hirano et al., 2008), which then provide a platform onto which the β-subunits incorporate (Figure 3; Frentzel et al., 1994; Nandi et al., 1997; Schmidtke et al., 1997). Initial assembly of the α-ring is controlled by two hetero-dimeric chaperones, termed Pba1-Pba2 and Pba3-Pba4 in yeast, and PAC1-PAC2 and PAC3-PAC4 in mammals, that provide scaffolds upon which the α-rings are built (Hirano et al., 2005; Kock et al., 2015; Wani et al., 2015). Pba1-Pba2 can associate with individual α-subunits *in vitro* and *in vivo* to initiate α-ring formation (Hirano et al., 2005; Le Tallec et al., 2007). Both chaperone subunits also contain a HbYX motif that allows them to bind and stabilize adjacent α-subunits as they associate (Kusmierczyk et al., 2011). The HbYX motif of Pba1 inserts into a pocket formed at the α_5_-α_6_ subunit interface, whereas that of Pba2 inserts at the α_6_-α_7_ interface, which together likely generate an α_5_-α_6_-α_7_ trimer (Kusmierczyk et al., 2011). How the α_1_, α_2_, α_3_ and α_4_ subunits are subsequently integrated is unknown, but a role for the Pba3-Pba4 chaperone is likely (see below). Although still viable, yeast cells lacking Pba1-Pba2 accumulate immature CP species containing structurally unstable α-rings, from which α_5_ and α_6_ readily dissociate (Wani et al., 2015), while mammalian cells with reduced levels of PAC1-PAC2 accumulate fewer complete α-rings (Hirano et al., 2005).

Through binding to the pockets between the α_5_, α_6_ and α_7_ subunits, Pba1-Pba2 also prevents premature association of CP assembly intermediates with the RP or other activating factors (Stadtmueller et al., 2012). In mature 26S proteasomes, one of the α_5_-α_6_ or α_6_-α_7_ pockets is occupied by the HbYX motif of Rpt5 (Tian et al., 2011; Beck et al., 2012; Schweitzer et al., 2016). Because Pba1-Pba2 appears to have a much higher affinity for α_5_-α_6_-α_7_ present in the CP intermediates as compared to those in the mature CP, they can outcompete Rpt5 and the rest of the RP for binding until the α-ring matures (Wani et al., 2015). It remains unclear what causes this affinity switch of Pba1-Pba2 for the α-ring, but allosteric effects caused by processing of the β-subunit propeptides, or steric alterations in the sizes of the α-ring pore and HbYX-binding pockets, might be involved (Kusmierczyk et al., 2011; Stadtmueller et al., 2012; Kock et al., 2015; Wani et al., 2015).

The Pba3-Pba4 heterodimer also participates in the early stages of α-ring assembly (Hirano et al., 2006, 2008; Le Tallec et al., 2007; Yashiroda et al., 2008). It binds tightly to the surface of the α_5_ subunit that faces the β-subunits (Kusmierczyk et al., 2008; Yashiroda et al., 2008), and is thus displaced from the ring by incoming β_4_ (Figure 3; Hirano et al., 2008). Pba3-Pba4 has a unique role among assembly chaperones in that it ensures formation of canonical 20S proteasomes in which each α-subunit is present in its correct position (Kusmierczyk et al., 2008). In the absence of Pba3-Pba4, aberrant α-subunit rings accumulate, containing an invariant α_5_-α_6_-α_7_-α_1_ hetero-tetramer, plus various arrangements of α_2_, α_3_ and α_4_ (Velichutina et al., 2004; Kusmierczyk et al., 2008; Takagi et al., 2014; Padmanabhan et al., 2016). Only in the presence of Pba3-Pba4 are all seven α-subunits integrated in correct register, thus generating a uniform CP architecture.

Upon completion, the α-ring provides a platform for assembling the β-ring, formation of which starts with β_2_, followed by sequential incorporation of the β_3_, β_4_, β_5_, β_6_ and β_1_ subunits (Figure 3). Entry of the “early” β subunits β_2_, β_3_ and β_4_ creates a semi-stable 13S intermediate (Li et al., 2007; Hirano et al., 2008), while subsequent entry of β_5_, β_6_ and β_1_ gives rise to a semi-stable 15S intermediate (Li et al., 2007; Hirano et al., 2008). In both yeast and mammals, β_7_ is the last β-subunit to integrate (Marques et al., 2007; Hirano et al., 2008; Li et al., 2016), leading to a transient species called the “half-proteasome.” Most β-subunits, excluding β_3_ and β_4_, are synthesized as precursors bearing an N-terminal propeptide, which helps with ring assembly and is then removed in mature particles. For example, the propeptides in β_2_ and β_5_ are essential for recruiting and incorporating β_3_ and β_6_, respectively, into the β ring (Chen and Hochstrasser, 1996; Hirano et al., 2008). For β_1_, β_2_, and β_5_, it is also critical that these extensions be removed to expose their N-terminal catalytic threonine residues that are essential for peptide bond cleavage (Chen and Hochstrasser, 1996; Schmidtke et al., 1996; Seemuller et al., 1996; Huber et al., 2016; Li et al., 2016).

Construction of the β-ring is also aided by binding of the Ump1 chaperone at the center of the α-ring prior to or concomitant with β_2_ binding (Figure 3; Ramos et al., 1998; Sá-Moura et al., 2013). Yeast lacking the intrinsically disordered Ump1 accumulate CP precursors, arguing that it plays a positive role in assembly (Ramos et al., 1998). However, genetic studies have implied a negative role, specifically by preventing premature dimerization of partially assembled α/β-ring precursors until a complete 15S half-proteasome is formed (Li et al., 2007). The N-terminal third of Ump1, which is dispensable for CP binding (Burri et al., 2000), performs this checkpoint function. The proximity of this region to β_6_ ideally positions Ump1 to both block dimerization and sense the arrival of β_7_ as the final subunit to be incorporated (Kock et al., 2015).

Integration of β_7_ promotes dimerization of two half-proteasomes by insertion of its C-terminal tail into a groove between β_1_ and β_2_ in the opposite β-ring (Figure 3). Following this coupling, the propeptides of β_1_, β_2_ and β_5_ undergo auto-catalytic cleavage to expose their N-terminal catalytic threonine. These active sites then proteolytically trim the neighboring propeptides of β_6_ and β_7_ (Chen and Hochstrasser, 1996; Schmidtke et al., 1996; Seemuller et al., 1996; Huber et al., 2016; Li et al., 2016). Ump1 remains bound through half-proteasome dimerization and β-subunit processing and ultimately becomes trapped inside the CP when assembly is complete. It is then degraded by the nascent β-subunit active sites, thus becoming the first substrate of each proteasome (Ramos et al., 1998; Burri et al., 2000; Griffin et al., 2000; Li et al., 2007; Hirano et al., 2008).

Finally, the CP is transiently capped with Blm10 (known as PA200 in plants and humans). This >200 kDa HEAT-repeat protein forms a dome on top of the CP (Schmidt et al., 2005; Sadre-Bazzaz et al., 2010) using its C-terminal HbYX motif for α-ring docking (Dange et al., 2011). Blm10 likely confers increased stability to the CP (Li et al., 2007; Lehmann et al., 2008). For example, when deletion of the β_7_ tail is combined with deletion of the *BLM10* gene, yeast cells exhibit a severe CP assembly defect (Marques et al., 2007). Additional functions have been ascribed to Blm10, including the potential to block entry of substrates into the CP lumen (Sadre-Bazzaz et al., 2010; Dange et al., 2011), promote CP import into the nucleus (Weberruss et al., 2013), and deliver dissociated CP into cytoplasmic proteasome storage granules (PSGs) in response to metabolic stress (Weberruss et al., 2013; Marshall and Vierstra, 2018b). In addition, CP-Blm10 complexes are particularly abundant upon treatment of cells with proteasome inhibitors (Marshall et al., 2015; Welk et al., 2016). Although the function(s) of these particles remain unknown, the association of Blm10 with the CP could reflect accelerated assembly of 26S proteasomes during such proteotoxic stress.

Although assembly of immuno- and thymo-proteasomes proceeds in a similar step-wise manner, the three catalytic subunits (LMP2, MECL1 and LMP7/PSMB11) are co-operatively and preferentially incorporated in place of their constitutive counterparts (β_1_, β_2_, and β_5_, respectively). One notable difference is that LMP2 enters the immuno-proteasome assembly pathway much earlier than for standard proteasomes, where β_1_ is typically the penultimate subunit to be incorporated (Li et al., 2007; Hirano et al., 2008). An intermediate complex is formed containing an α-ring, LMP2, MECL1, β_3_ and β_4_ (Nandi et al., 1997), with LMP2 and MECL1 being incorporated simultaneously in a mutually dependent manner (Groettrup et al., 1997; Griffin et al., 1998; Kingsbury et al., 2000). LMP7 is then recruited preferentially over β_5_ into LMP2- and MECL1-containing intermediates (Griffin et al., 1998; Kingsbury et al., 2000). LMP7 binds more tightly to POMP/UMP1 than β_5_, and can incorporate independently of β_4_ (Bai et al., 2014), both of which promote immuno-proteasome assembly. The inter-dependency of LMP2 and MECL1 incorporation typically results in assembly of homogenous immuno- and thymo-proteasomes that contain all three inducible subunits (Kingsbury et al., 2000). These variants amass approximately four times faster than standard proteasomes (Heink et al., 2005), enabling a rapid response to immune and inflammatory stimuli.

## Regulated Assembly of the Proteasome Regulatory Particle

Unlike the CP, which is composed entirely of ring structures, the RP is more architecturally heterogeneous, with the base and lid sub-complexes assembling independently of each other (Lander et al., 2012; Beckwith et al., 2013; Tomko and Hochstrasser, 2014; Tomko et al., 2015). As with the CP, the RP base depends heavily on dedicated assembly chaperones for correct positioning for the six members of the Rpt ring (Figure 3). Thus far, four Rpt chaperones have been described: Nas2, Nas6, Hsm3 and Rpn14 in yeast, known as p27, p28, S5b, and PAAF1, respectively, in mammals (Funakoshi et al., 2009; Kaneko et al., 2009; Le Tallec et al., 2009; Park et al., 2009; Roelofs et al., 2009; Saeki et al., 2009). These chaperones are unrelated in sequence and independently bind to the C-terminal domain of a distinct Rpt subunit, resulting in the formation of three precursor assembly modules: Nas2-Rpt4-Rpt5, Nas6-Rpt3-Rpt6-Rpn14, and Hsm3-Rpt1-Rpt2-Rpn1 (Figure 3; Lee S. Y. et al., 2011; Barrault et al., 2012; Takagi et al., 2012; Park et al., 2013; Satoh et al., 2014). These modules are stabilized in part by the intertwining N-terminal coiled-coil regions of the Rpt subunit pairs (Zhang et al., 2009), which at least for one pair (Rpt1-Rpt2) is thought to begin co-translationally (Panasenko et al., 2019). As described below, the Nas2 and Nas6 modules first associate with each another, followed by incorporation of the Hsm3 module, along with Rpn2 and Rpn13. Rpn10 is then recruited to complete assembly of the RP base. A checkpoint involving ubiquitylation of Rpt5 by the RING E3 Not4 helps ensure that the chaperone-bound modules are integrated in the correct order (Fu et al., 2018).

Currently, two mutually non-exclusive routes for base assembly have been proposed; in the first, the base assembles alone, whereas in the second, base assembly is templated by the CP. The first model is supported in yeast by the detection of fully-constructed base sub-complexes containing assembly chaperones, coupled with the absence of these chaperones in holo-26S proteasomes (Kriegenburg et al., 2008; Funakoshi et al., 2009; Le Tallec et al., 2009; Park et al., 2009; Roelofs et al., 2009; Saeki et al., 2009). Immunoprecipitation experiments then showed that Nas2 readily co-purifies with all components of the Nas2 and Nas6/Rpn14 modules, but not with components of either the Hsm3 module, lid, or CP (Tomko et al., 2010). An analogous stepwise incorporation was inferred in mammalian cells (Kaneko et al., 2009), although the Nas2 module, rather than the Hsm3 module, was proposed to be the last to enter the emerging RP base. Fully constructed base sub-complexes complete with chaperones could also be achieved in *E. coli* by co-expressing the nine base subunits along with the four constitutive base assembly chaperones (Beckwith et al., 2013). As *E. coli* is devoid of proteasomes and associated proteins, this recombinant system defined the minimal environment for base assembly and provided unequivocal evidence that the RP base can self-organize independently of the CP and RP lid.

In the templated model of base assembly, base modules are delivered to the CP and connected directly on the surface of the CP α-ring. This model originated from the detection of base assembly intermediates associated with the CP when the α-ring was compromised (Kusmierczyk et al., 2008). Additionally, C-terminal truncations of Rpt4 and Rpt6 created strong base assembly defects, suggesting that docking of the C-terminal HbYX motifs in these subunits onto the CP is critical for base assembly *in vivo* (Park et al., 2009). Both models agree that chaperones must dissociate from the assembled base to properly dock the RP onto the CP to then trigger gate opening. The base appears to exploit ATP-dependent conformational changes in the Rpt subunits to evict the chaperones and allow stable RP-CP association (Roelofs et al., 2009; Park et al., 2013). This mechanism was recently described in detail for Nas6 (Nemec et al., 2019); upon lid-base association, interaction of Rpn5 with the base promotes an ATP-dependent conformational change in Rpt3 that drives release of Nas6 from the nascent proteasome.

Recently, Adc17 was identified as an adaptive proteasome assembly chaperone that regulates the Nas6-Rpt3-Rpt6-Rpn14 module in yeast (Hanssum et al., 2014). Adc17 associates with the N-terminal domain of Rpt6 and appears to promote Rpt3-Rpt6 dimerization, which in turn enhances proteasome assembly under conditions that elicit proteotoxic stress. Expression of Adc17 is induced under these conditions via a mechanism independent of Rpn4 but regulated by the central stress and autophagy regulator Tor1/2 (Hanssum et al., 2014). Pharmacological or genetic inhibition of Tor1/2 enhances expression of Adc17 (and other proteasome assembly chaperones) via the mitogen-activated protein kinase Mpk1 (ERK5/MAPK7 in mammals; Rousseau and Bertolotti, 2016), thus representing a novel route for up-regulating 26S proteasome assembly when its capacity is exceeded.

Co-expression studies imply that RP lid biogenesis begins with dimerization of Rpn8 and Rpn11, followed by recruitment of Rpn6 (Estrin et al., 2013), which then conscripts Rpn5 and Rpn9 to the particle (Sharon et al., 2006). In parallel, Rpn3 and Rpn7 are brought together by Sem1 to form a hetero-trimeric complex (Figure 3; Fukunaga et al., 2010; Tomko and Hochstrasser, 2011, 2014). These two sub-complexes then combine to create a nearly complete lid intermediate that lacks only Rpn12, which becomes the final subunit to associate (Fukunaga et al., 2010; Tomko and Hochstrasser, 2011; Tomko et al., 2015). While no assembly chaperones have yet been identified for the RP lid, the unusual proteasome subunit Sem1 likely plays a critical role (Tomko and Hochstrasser, 2014). Sem1 escaped detection for many years because of its small size, near-complete lack of secondary and tertiary structure, and an absence of lysine residues that challenged its detection by proteomic methods (Russell et al., 2013; Kragelund et al., 2016). Well-resolved cryo-EM views have since shown that it binds to a hydrophobic pocket between Rpn3 and Rpn7 to stabilize an otherwise weak interaction during the early stages of lid biogenesis (Wei et al., 2008; Tomko and Hochstrasser, 2014; Dambacher et al., 2016).

It is also becoming clear that Rpn12 is pivotal to lid maturation by inducing several conformational changes upon integration (Estrin et al., 2013; Tomko et al., 2015). The RP intermediate lacking Rpn12 adopts a more compact state as compared to that found in the complete RP and, surprisingly, introduction of just the C-terminal α-helix of Rpn12 is sufficient to drive this large-scale conformational re-organization (Tomko et al., 2015). The Rpn12 α-helix sits centrally within a helical bundle created by clustering of the C-termini of most Rpn subunits, and thus might be responsible for “sensing” the assembly state of the lid.

The Rpn8-Rpn11 deubiquitylating module also undergoes a conformational change during lid maturation (Dambacher et al., 2016). In the isolated lid, this module is positioned perpendicular to its orientation in the holo-proteasome, which is likely incompatible with base binding and, importantly, might auto-inhibit the deubiquitylating activity of Rpn11 until RP assembly is complete (Tomko et al., 2015; Dambacher et al., 2016). It also remains possible that additional motions beyond those involving Rpn12 and Rpn8-Rpn11 are necessary for the lid-base connection.

The final step in 26S proteasome assembly is association of the RP with the CP (Figure 3). Binding is driven by docking of the C-terminal HbYX motifs from several Rpt ring subunits into pockets between adjacent CP α-subunits, which also promotes gate opening and substrate entry into the CP lumen (Smith et al., 2005, 2007; Rabl et al., 2008; Tian et al., 2011; Park et al., 2013). This association occurs spontaneously *in vitro* (Liu et al., 2006; Livnat-Levanon et al., 2014), is stabilized by ATP (Smith et al., 2005; Liu et al., 2006), and is fully reversible (Bajorek et al., 2003; Kleijnen et al., 2007; Wang et al., 2010b; Marshall and Vierstra, 2018b). Rpn6 is thought to help tether the RP to the CP through binding to the α_2_ subunit (Lander et al., 2012; Pathare et al., 2012). Several additional factors have also been implicated, including Ecm29, which appears to provide a critical quality control checkpoint by binding to structurally aberrant proteasomes and repressing both the ATPase activity of the RP and gate opening of the CP in these particles (Lehmann et al., 2010; Lee S. Y. et al., 2011; Panasenko and Collart, 2011; Park et al., 2011; De La Mota-Peynado et al., 2013; Wang et al., 2017). Hsp90 has also been implicated in CP-RP assembly (Imai et al., 2003; Yamano et al., 2008), but its precise role(s) remain unclear.

At present, there is only a rudimentary understanding of 26S proteasome assembly in plants. Proteasome preparations from *Arabidopsis* routinely contain free CP, RP, and singly- and doubly-capped 26S particles, along with a definitive relative of Blm10 (PA200) connected to the CP (Yang et al., 2004; Book et al., 2010). Mutants eliminating PA200 do not display defects in phenotype, ubiquitin conjugate accumulation, proteasome activity, or sensitivity to proteasome inhibitors (Book et al., 2010). However, a role for PA200 in proteasome regulation is inferred by its ability to bind to the CP under conditions that induce proteotoxic stress (Book et al., 2010; Marshall et al., 2015), like its mammalian counterpart (Welk et al., 2016). PA200 is also essential for the entry of free CPs into PSGs during fixed-carbon starvation, and thus has a role in proteasome storage (Marshall and Vierstra, 2018b; see below). Possible orthologs of the yeast assembly chaperones, Pba1, Pba2, Pba3, Pba4, Ump1, Nas2, Nas6, Hsm3, and Ecm29 have also been detected in plants, but their amino acid sequence similarities are sufficiently low to prevent conclusive assignments (D. C. Gemperline, R. S. Marshall, and R. D. Vierstra, unpublished data). However, the expression of most, if not all, of these putative chaperones is up-regulated upon proteasome inhibition in *Arabidopsis* (Gladman et al., 2016), as might be expected for factors needed to assemble proteasomes when supply is limited.

## Subcellular Localization of 26S Proteasomes

**Figure 4:**
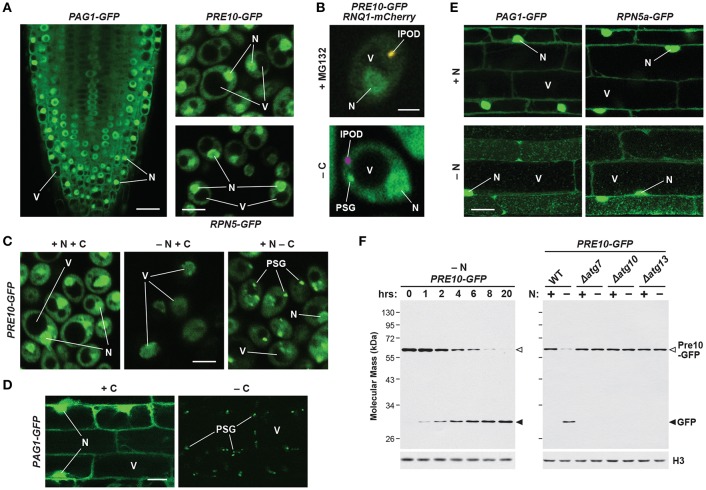
Intracellular Localization of 26S Proteasomes in *Arabidopsis* and Yeast. The location of proteasomes was tracked by tagging proteasome subunits with GFP, which allows *in vivo* detection via confocal fluorescence microscopy **(A–E)**, and a quantitative assay for proteaphagy by measuring the release of free GFP from the tagged subunits upon entry into vacuoles **(F)**. **(A)** 26S proteasomes are found in the cytosol and nucleus of *Arabidopsis* and yeast cells grown in nutrient-rich conditions. Shown is localization of the PAG1-GFP protein in root tip cells of a 7-day-old *Arabidopsis* seedling (left), or the Pre10-GFP or Rpn5-GFP proteins in exponential phase yeast cells (top right and bottom right, respectively). Scale bars, 25 μm (left) and 1 μm (right). **(B)** Yeast 26S proteasomes localize into IPOD-like structures upon inhibition, but to PSGs upon carbon starvation. Cells expressing Pre10-GFP and the IPOD marker Rnq1-mCherry were grown in nutrient-rich medium then switched to either medium containing 80 μM MG132 (top) or medium lacking carbon (bottom) for 8 h and imaged by confocal fluorescence microscopy. Scale bar, 1 μm. **(C)** Yeast 26S proteasomes are delivered to the vacuole upon nitrogen starvation but sequester into cytoplasmic PSGs upon carbon starvation in yeast. Cells expressing Pre10-GFP were grown in nutrient-rich medium, switched to medium lacking either nitrogen or carbon for 8 h, and then imaged by confocal fluorescence microscopy. Scale bar, 1 μm. **(D)** 26S proteasomes are sequestered into cytoplasmic PSGs upon fixed carbon starvation in *Arabidopsis*. 7-day-old *Arabidopsis* seedlings expressing PAG1-GFP were grown in the light in sucrose-containing medium and then switched to growth in the dark in sucrose-free medium for 16 h. Root cells of the lower elongation zone were imaged by confocal fluorescence microscopy. Scale bar, 10 μm. **(E)** *Arabidopsis* 26S proteasomes are sequestered in autophagic bodies inside vacuoles upon nitrogen starvation. Seedlings expressing PAG1-GFP or RPN5a-GFP were grown on nutrient-rich medium and then switched to growth on nitrogen-free medium plus 1 μM concanamycin A for 16 h. Root cells of the lower elongation zone were imaged by confocal fluorescence microscopy. Scale bar, 10 μm. **(F)** Time course for the autophagy-mediated release of free GFP from Pre10-GFP upon nitrogen starvation in yeast. Wild-type (WT) or autophagy-defective Δ*atg7*, Δ*atg10*, or Δ*atg13* cells expressing Pre10-GFP were grown in nutrient-rich medium then switched to medium lacking nitrogen for the indicated times (left panel) or 8 h (right panel). Total protein extracts were then assayed for accumulation of free GFP by immunoblot analysis with anti-GFP antibodies. Open and closed arrowheads locate the Pre10-GFP fusion and free GFP, respectively. Immunodetection of histone H3 was used to confirm near-equal protein loading. In panels **(A–E)**; N, nucleus; V, vacuole; IPOD, insoluble protein deposit; PSG, proteasome storage granule. Images were adapted with permission from Marshall et al. (2015, 2016) and Marshall and Vierstra (2018b).

Fully assembled 26S proteasomes are not static entities, but instead exhibit dynamic behavior by dissociating into free RP and CP sub-particles, shuttling between the cytoplasm and nucleus, and re-locating between compartments in response to different growth, development or environmental challenges. When tagged with GFP, most proteasome subunits fully incorporate into their appropriate sub-complexes, thus enabling live cell imaging of the CP, RP, and/or holo-26S particles. Using these reporters in yeast, mammals and plants, it is evident that the CP and RP are diffusely spread throughout both the cytosol and nucleus, though often substantially enriched in the latter compartment (Figure 4A; Reits et al., 1997; Enenkel et al., 1998; Russell et al., 1999; Brooks et al., 2000; Pack et al., 2014; Marshall et al., 2015). Measurements of proteasome activity in the two compartments have varied greatly (Gardner et al., 2005; Chen and Madura, 2014; Dang et al., 2016). Numerous studies, including recent cryo-electron tomographic imaging in the green alga *Chlamydomonas reinhardtii*, found that proteasomes are not distributed evenly within the nucleus, but instead accumulate at the inner nuclear membrane, in the vicinity of nuclear pore complexes (Enenkel et al., 1998; Takeda and Yanagida, 2005; Albert et al., 2017).

Fluorescence correlation spectroscopy determined the absolute concentration of the 26S proteasome in actively dividing yeast cells to be 830–980 nM in the nucleus but only 140–200 nM in the cytoplasm (Pack et al., 2014), with similar concentrations observed in cultured mammalian neuronal cells (Asano et al., 2015). However, proteasome concentration can be much higher in localized areas at the inner nuclear membrane, being recorded at over 8 μM in *C. reinhardtii* (Albert et al., 2017). By contrast, proteasomes in quiescent cells are exported from the nucleus and sequestered into reversible, motile cytoplasmic PSGs that collectively reflect a rapid and dramatic re-localization of 26S proteasomes out of the nucleus, presumably for storage (Figures 4B–D; Bingol and Schuman, 2006; Laporte et al., 2008; Yedidi et al., 2016; Gu et al., 2017; Marshall and Vierstra, 2018b). Re-feeding with a fresh carbon source immediately reverses this process by stimulating rapid import of the RP and CP sub-particles back into the nucleus followed by holo-26S proteasome assembly. While not found in granules, aged proteasomes (over 3 days old) were similarly found to be largely cytosolic in mouse embryonic fibroblasts (Tomita et al., 2019).

Given the sheer size of 26S proteasomes and their RP and CP sub-particles, a major challenge to cells during proteasome re-localization is the transport of these particles into and out of the nucleus through their size-limited nuclear pores (Beck and Hurt, 2017). In proliferating yeast, proteasomes are imported into the nucleus as CP and RP assembly intermediates, each of which bears one or more nuclear localization signals (NLS; Tanaka et al., 1990; Nederlof et al., 1995). The NLS is recognized by an importin-α/β heterodimer assembled from two members of the β-karyopherin family, termed Srp1/Kap60 and Kap95, respectively (Enenkel et al., 1995). Given that only a small number of proteasome subunits contain an NLS, it was originally speculated that yeast proteasomes enter the nucleus as separate CP, RP lid and RP base sub-complexes (Lehmann et al., 2002; Wendler et al., 2004; Isono et al., 2007). However, several studies subsequently implied that the final steps of CP assembly occur in the nucleus after importin-α/β dependent transport. For example, co-immunoprecipitation studies with Srp1 detected its association with CP assembly intermediates but not with the mature CP, as reflected by the presence of unprocessed β_5_ subunit propeptides (Lehmann et al., 2002). Additionally, yeast CP assembly intermediates accumulate in the nucleus when their maturation is suppressed by deletion of Ump1 (Lehmann et al., 2002).

The CP has been proposed to exist in import-competent and import-incompetent configurations, depending on accessibility of the NLS within specific α-subunits (Tanaka et al., 1990). Recent cryo-EM structures support this hypothesis by showing that NLS sequences in the CP are exposed in assembly intermediates due to disorder within the α-rings (Kock et al., 2015; Wani et al., 2015), but are masked in more mature particles due to conformational changes that close the α-rings and permit RP binding. In a similar fashion, the RP base appears to be imported by itself into the nucleus using an NLS within the Rpt2 or Rpn2 subunits that binds importin-α/β (Wendler et al., 2004; Isono et al., 2007; Savulescu et al., 2011; Weberruss et al., 2013). Blm10, a protein structurally related to Rpn2, also facilitates nuclear import of mature CP upon resorption of PSGs, when quiescent cells resume growth following periods of starvation (Weberruss et al., 2013).

A collection of studies also indicate that entire holo-proteasomes can undergo nuclear translocation without disassembly (Reits et al., 1997; Chen et al., 2011; Savulescu et al., 2011; Pack et al., 2014). This should be possible given that the channel of the nuclear pore complex can expand to accommodate cargo with a diameter of up to 39 nm (Pante and Kann, 2002), although the mechanism by which this might occur remains obscure (Burcoglu et al., 2015). The most convincing evidence comes from a genetically stabilized 26S proteasome in which the α_4_ subunit of the CP was translationally fused to the Rpt1 or Rpt2 subunits of the RP, thus blocking CP-RP dissociation. Surprisingly, these 26S proteasomes did not exhibit obvious structural defects and were distributed normally in the nucleus, even upon exit of cells from stationary phase when cytosolic PSGs dissolve and the levels of nuclear proteasomes returned back to normal (Laporte et al., 2008; Pack et al., 2014). Since protein synthesis is stalled during quiescence, CP precursors were not available for import, leading to the conclusion that a nuclear import pathway exists that makes use of the older, mature, stabilized complexes (Pack et al., 2014). As will be described below, nuclear 26S proteasomes also become substrates of autophagy following nitrogen starvation or inactivation, which *a priori* requires export from the nucleus. A current model posits that 26S particles dissociate into free, stable CP and RP sub-complexes, which are then separately exported (Nemec et al., 2017).

In addition to nuclear and cytoplasmic proteasomes, a plasma membrane-localized form of the CP was recently described in mammalian neurons (Ramachandran and Margolis, 2017). This novel CP is exposed to the cell surface, and appears to exclusively degrade ribosome-associated nascent polypeptides in a ubiquitin-independent manner upon their synthesis after neuronal stimulation (Ramachandran et al., 2018). An intriguing possibility is that these bound proteasomes directly extrude peptides out of the cell to attenuate neuronal activity-induced calcium signaling (Ramachandran and Margolis, 2017). Whether such membrane-associated proteasomes exist in other organisms or cell types remains to be determined.

## Proteasome Regulation by Post-Translational Modification

Post-translational modifications of 26S proteasomes offer additional opportunities to influence proteasome assembly, activity, localization and abundance. Thus far, over 350 sites of post-translational modification have been identified on the 26S particle, which include acetylation, ADP-ribosylation, glycosylation, methylation, myristoylation, oxidation, phosphorylation, SUMOylation, ubiquitylation, and proteolytic processing (Kikuchi et al., 2010; Cui et al., 2014; Hirano et al., 2016). In fact, the same proteasome site might be targeted by more than one modification, suggesting cross-talk between different types (Zong et al., 2014). Unfortunately, the functional consequences for most of these alterations are currently unclear.

One common modification is phosphorylation, which affects almost all proteasome subunits and is directed by an assortment of proteasome-interacting kinases and phosphatases (Iwafune et al., 2002; Lu et al., 2008; Kikuchi et al., 2010). As an example of the importance of phosphorylation, treatment of purified mammalian proteasomes with alkaline phosphatase leads to dissociation of the CP and RP (Satoh et al., 2001). Phosphorylation of Ser-120 in RPT6 by protein kinase A (PKA), and its dephosphorylation by protein phosphatase 1γ (PP1γ), likely regulates the interaction between RPT6 and the α_2_ subunit of the CP to effect this dissociation (Satoh et al., 2001; Asai et al., 2009). Ser-14 of RPN6 also becomes phosphorylated by PKA, which leads to increased levels of doubly-capped proteasomes, thus stimulating overall protein degradation rates (Lokireddy et al., 2015), consistent with the proposed role for RPN6 in mediating CP-RP association (Lander et al., 2012; Pathare et al., 2012). Another example is the phosphatase UBLCP1, which binds to RPN1 via a UBL domain and subsequently dephosphorylates RPT1. This modification regulates nuclear proteasome assembly, again by controlling association of the RP and CP (Guo et al., 2011; Sun et al., 2017). The interaction of Ecm29 with the proteasome is similarly regulated by phosphorylation of the CP subunit α_7_ (Wani et al., 2016).

Ubiquitylation of 26S proteasomes has been shown to have multiple effects. Extensive ubiquitylation of the yeast and *Arabidopsis* particles directs non-functional complexes for autophagic degradation via specific receptors that bind to both the ubiquitin moieties on the impacted proteasome subunits and ATG8 (Marshall et al., 2015, 2016; Cohen-Kaplan et al., 2016; see below). As mentioned above, specific ubiquitylation of the proteasomal ubiquitin receptors Rpn10 and Rpn13 suppresses their ability to recognize substrates (Isasa et al., 2010; Lipinszki et al., 2012; Jacobson et al., 2014; Zuin et al., 2015), while ubiquitylation of Rpt5 appears to be an important checkpoint during Rpt ring assembly (Fu et al., 2018).

The function(s) of other 26S proteasome modifications are less well-defined. The Rpt2 subunit of the RP has been shown to be N-myristoylated in multiple species, which could tether proteasomes to membrane surfaces (Shibahara et al., 2002; Gomes et al., 2006; Kimura et al., 2012, 2016). In yeast, the N-terminus of Rpt1 is mono- or di-methylated, and a mutant strain blocking this modification is more sensitive to proteotoxic stress induced by hydrogen peroxide or the amino acid analog canavanine (Kimura et al., 2013). Other examples include glutathionylation of the α_5_ subunit, which might affect gating of the yeast CP (Demasi et al., 2003; Silva et al., 2012), and attachment of N-acetylgalactosamine to mammalian RPT2, which inhibits the ATPase ring of the RP base and hence reduces overall proteasome degradation rates (Zhang et al., 2003). A role for N-acetylation of proteasome subunits by the NatB complex in assembling PSGs has been inferred from the effects of Δ*nat3* and Δ*mdm20* mutants on this re-localization (van Deventer et al., 2015; Marshall and Vierstra, 2018b). Further work is clearly needed to establish the reasons for the myriad of other modifications.

## Autophagy-Mediated Control of 26S Proteasome Abundance

While the synthesis and assembly of proteasomes has been studied for over a decade, their turnover had remained obscure until recently. Proteasomes are stable complexes (Pack et al., 2014), with a half-life of 16 h in mouse embryonic fibroblasts (Tomita et al., 2019) and over 2 weeks when measured in rat liver cells (Tanaka and Ichihara, 1989), but under specific conditions their degradation can be rapid and extensive. One turnover mechanism involves caspase-mediated cleavage. Following induction of apoptosis in human cells, the RP subunits RPT5, RPN2 and RPN10 are cleaved by caspase-3, resulting in impaired proteasome activity and the accumulation of ubiquitylated substrates (Sun et al., 2004). Similarly, caspase-3 activation in *D. melanogaster* cells leads to cleavage of the α_2_, α_4_ and β_4_ subunits of the CP, and the RPT1 subunit of the RP (Adrain et al., 2004). Presumably these impaired proteasomes are then removed, possibly by autophagy (see below). A second pathway is the removal of non-functional proteasome subunits by the UPS itself prior to their integration into the holo-proteasome. Hsp42 was shown to be important in yeast by coalescing these polypeptides into cytoplasmic condensates from which they are cleared by active 26S proteasomes (Peters et al., 2015; Nahar et al., 2019).

**Figure 5:**
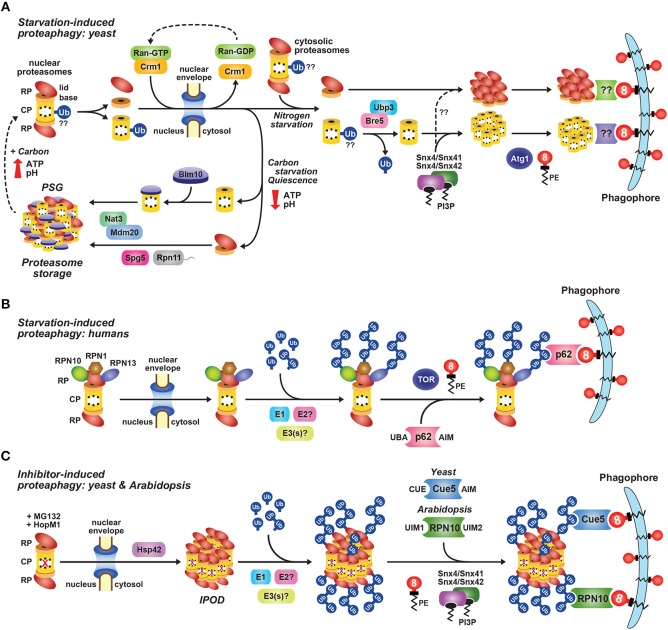
Pathways for Autophagic Degradation of 26S Proteasomes. **(A)** A model for starvation-induced proteasome degradation versus storage in yeast. When cells are subjected to nitrogen or carbon starvation, 26S proteasomes dissociate into the CP and RP sub-complexes and are exported from the nucleus via the exportin Crm1. Upon nitrogen starvation, the CP and RP coalesce into cytoplasmic foci in a Snx4/41/42-dependent manner. They are then encapsulated by the expanding phagophore and delivered to the vacuole for degradation, a process requiring the Atg1 kinase complex and the Atg8 lipidation machinery. Deubiquitylation of one or more CP subunits by Ubp3/Bre5 may also be required. Whether specific Atg8-binding autophagy receptors are involved remains unknown. In contrast, carbon starvation, which results in cytoplasmic acidification and reduced ATP levels, triggers re-localization of the CP and RP into cytoplasmic proteasome storage granules (PSGs). This accretion requires numerous factors, including Blm10 for the CP, Spg5 and the C-terminus of Rpn11 for the RP, and the NatB N-terminal acetylation complex (consisting of Nat3 and Mdm20) for both. PSGs act to store proteasome sub-complexes and protect them from autophagic degradation. Preventing sequestration of proteasomes into PSGs leads to their Atg1- and Atg8-dependent turnover. **(B)** A model for starvation-induced proteasome degradation in humans. When HeLa cells are subjected to amino acid starvation, the proteasome subunits RPN1, RPN10, and RPN13 become poly-ubiquitylated by one or more E3 ligases, facilitating their recognition by the autophagy receptor p62/SQSTM1. By simultaneous interaction with lipidated ATG8/LC3, p62 delivers inactive proteasomes to the expanding phagophore for eventual turnover by autophagy, a process requiring the TOR kinase and the ATG8/LC3 lipidation machinery. **(C)** A model for inhibitor-induced proteaphagy in *Arabidopsis* and yeast. Proteasomes subjected to chemical or genetic inhibition, including by the pathogen effector HopM1, are exported from the nucleus and aggregate in an Hsp42-dependent manner into insoluble protein deposit (IPOD)-like structures that are distinct from PSGs. The aggregated proteasomes are then ubiquitylated by one or more E3 ligases, facilitating their recognition by the selective proteaphagy receptors Cue5 in yeast or RPN10 in *Arabidopsis*. By simultaneous interactions with lipidated ATG8, these receptors deliver inactive proteasomes to enveloping autophagic vesicles for final turnover in the vacuole. PE, phosphatidylethanolamine; PI3P, phosphatidylinositol-3-phosphate.

**Figure 6:**
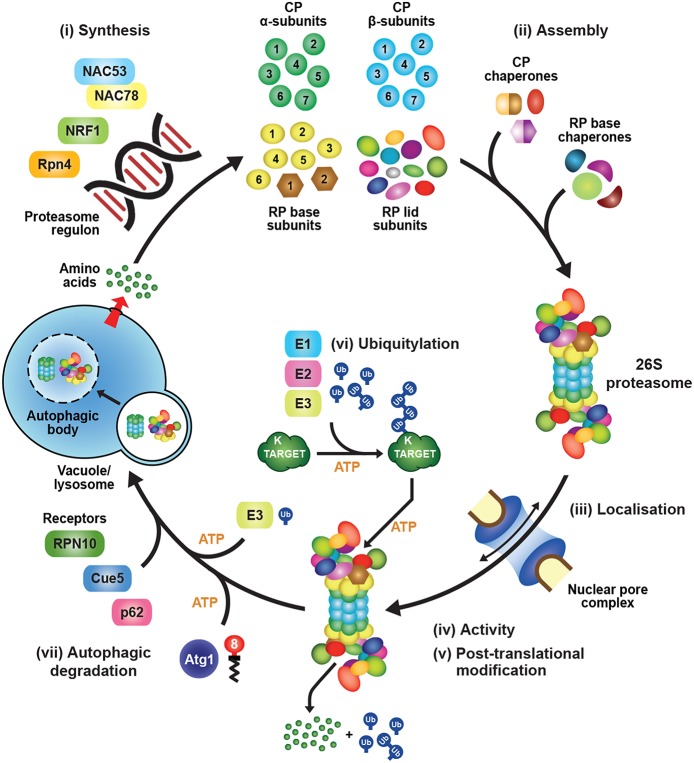
The Life Cycle of a 26S Proteasome. Proteasome subunit synthesis from individual amino acids is regulated by transcription factors such as Rpn4 in yeast, NRF1 in mammals, and NAC53/NAC78 in plants, the activities of which are sensitive to changing physiological conditions, in particular proteotoxic stress. The various subunits then assemble in a co-ordinated manner to form the mature holo-26S proteasome, with assistance from a suite of dedicated chaperones. Proteasomes localize to either the cytosol or nucleus, where their activity can be regulated by an array of post-translational modifications and associated factors. They ultimately recognize and degrade poly-ubiquitylated substrates in a process mediated by intrinsic and extrinsic ubiquitin receptors. Finally, excess or damaged proteasomes can be degraded in the vacuole or lysosome via one of several autophagic pathways, some which are mediated by signals from the nutrient-responsive Atg1 kinase, subunit ubiquitylation, and/or a variety of autophagy receptors, including Cue5 in yeast, p62/SQSTM1 in mammals, and RPN10 in plants. Autophagic degradation of 26S proteasomes recycles amino acids, which can then be used for the synthesis of new particles.

A third pathway for degrading 26S proteasomes that has recently gained in appreciation is autophagy, via a route termed proteaphagy (Figure 5; Marshall and Vierstra, 2015; Marshall et al., 2015, 2016). Autophagy involves the delivery of cytoplasmic material to the vacuole (in plants and yeast) or lysosome (in mammals) for breakdown by resident hydrolases (Reggiori and Klionsky, 2013; Gatica et al., 2018; Marshall and Vierstra, 2018a; Levine and Kroemer, 2019). It is the preferred catabolic route for large, heterogeneous cytoplasmic material, such as protein aggregates, organelles, lipid droplets, or even invading pathogens whose sizes exceed the spatial capacity of proteasomes. The defining feature of the most common autophagic route, macroautophagy (referred to here as autophagy), is the *de novo* formation of a cup-shaped membrane called the phagophore (or isolation membrane) that encircles portions of cytoplasm. The phagophore ultimately seals to generate a double membrane-bound autophagosome, the outer membrane of which then fuses with the vacuole or lysosome to release the internal vesicle as an autophagic body (see Figure 6). The contents of the autophagic body and its limiting membrane are rapidly consumed by a collection of vacuolar hydrolases with acidic pH optima (Parzych and Klionsky, 2018), with the constituent amino acids, fatty acids, carbohydrates and nucleotides ultimately re-used for survival or to power new growth.

Through studies on a variety of organisms over the past two decades, the core machinery underpinning autophagy has emerged, driven by a conserved collection of “autophagy-related” (Atg) proteins. These are traditionally classified into distinct biochemical and functional groups that act at specific stages during autophagy, and include: (i) the Atg1 serine/threonine kinase complex that initiates autophagy in response to upstream signals from nutrient-sensitive kinases, such as Snf1 and Tor1/2; (ii) the Atg9 transmembrane protein required for membrane delivery; (iii) the class III phosphatidylinositol-3-kinase (PI3K) complex that generates the phosphatidylinositol-3-phosphate (PI3P) signal important for autophagosome nucleation; (iv) the Atg2-Atg18 complex involved in membrane extension at the site of PI3P labeling; and (v) the ubiquitin-fold protein Atg8 and its conjugation machinery that are crucial for autophagosome dynamics and cargo recruitment (Ohsumi, 2001; Marshall and Vierstra, 2018a; Levine and Kroemer, 2019).

Atg8 (known as MAP1LC3 or GABARAP in mammals) is the signature element of the autophagy system. Its functions depend on attachment to the lipid phosphatidylethanolamine (PE) via a conjugation cascade mechanistically analogous to ubiquitylation. Atg8 is activated by the E1 Atg7, transferred to the E2 Atg3, and finally connected via an ether linkage to PE by a hexameric E3 ligase complex comprised of a conjugate between Atg5 and Atg12 which is then bound to Atg16.

Lipidated Atg8 becomes embedded in the autophagic membranes, where it serves two purposes. One is to promote membrane expansion, autophagosome closure, and final docking with the vacuole or lysosome through interactions with a collection of adaptors that bind both components of the vesicular transport machinery and the Atg8-PE adduct. The other is to tether cargo to the enveloping phagophore through interactions between Atg8-PE and a plethora of receptors that recognize specific cargo (Rogov et al., 2014; Farré and Subramani, 2016; Gatica et al., 2018; Marshall and Vierstra, 2018a). The best-known adaptors/receptors bind Atg8 with low micromolar affinity through an Atg8-interacting motif [AIM, also called an LC3-interacting region (LIR)] bearing two hydrophobic residues that insert into complementary hydrophobic pockets on the surface of Atg8 (Noda et al., 2008, 2010; Klionsky and Schulman, 2014; Maqbool et al., 2016; Rogov et al., 2018), although additional binding mechanisms have recently been described (Marshall et al., 2019).

Through a rapidly expanding collection of receptors, a wide array of selective autophagic routes have emerged, including dedicated pathways for clearing protein aggregates, stress granules, mitochondria, peroxisomes, chloroplasts, ER, nuclear components, lipid bodies, ribosomes, and intracellular pathogens (Kraft et al., 2008; Mochida et al., 2015; Farré and Subramani, 2016; Khaminets et al., 2016; Yamano et al., 2016; Gatica et al., 2018; Marshall and Vierstra, 2018a; Wyant et al., 2018). As will be described below, proteasomes are also rapidly cleared by autophagy using at least two proteaphagic routes (Marshall and Vierstra, 2015; Marshall et al., 2015, 2016; Cohen-Kaplan et al., 2016; Waite et al., 2016; Nemec et al., 2017).

## Autophagic Degradation of 26S Proteasomes Upon Nutrient Starvation

Autophagic flux is up-regulated upon nutrient starvation, which includes lack of nitrogen, fixed-carbon, phosphate, and various micronutrients such as zinc, resulting in the bulk degradation of cytoplasmic material, often in a non-specific manner (Takeshige et al., 1992; Thompson et al., 2005; Adachi et al., 2017; Kawamata et al., 2017). Early hints that 26S proteasomes could be targets for autophagic degradation came from immuno-electron microscopy studies that observed 20S proteasome subunits in rat liver lysosomes, particularly upon starvation (Cuervo et al., 1995). Subsequently, multiple proteomic studies cataloging autophagosome contents identified proteasome subunits as cargo (Gao et al., 2010; Dengjel et al., 2012; Mancias et al., 2014; Le Guerroué et al., 2017), while more recent multi-omics studies in humans and maize confirmed that proteasomes are extensively degraded by both basal and starvation-induced autophagy (Zhang et al., 2016; McLoughlin et al., 2018).

Autophagic degradation of proteasomes can easily be visualized using subunits tagged with GFP or other fluorescent reporters. For example, transfer of the fluorescent signals from the nucleus and cytoplasm to autophagic bodies in the vacuole is evident within hours of nitrogen starvation in both *Arabidopsis* and yeast, and within 12 h almost all proteasomes from both organisms have moved to the vacuole via autophagy (Figures 4C,E). This transfer and subsequent breakdown can then be quantified by immunoblot analysis of the proteasome subunits fused to GFP-type reporters (Figure 4F). Whereas, the tagged proteasome subunit is rapidly degraded as the autophagic body breaks down, the GFP moiety is remarkably stable and accumulates in the vacuole. The ratio of the GFP fusion to free GFP thus provides a reliable assay to measure autophagic turnover rates. Using this assay in yeast, it was shown that more than 80% of cellular proteasomes are degraded by autophagy after 8 h of nitrogen starvation (Figure 4F; Marshall et al., 2016).

The starvation-induced autophagic degradation of proteasomes, along with similarly abundant ribosomes (Kraft et al., 2008; Wyant et al., 2018), will rapidly provide a pool of free amino acids that can sustain cell viability when nitrogen is scarce. Given the fast induction of autophagy when nutrients are limiting (Takeshige et al., 1992; Thompson et al., 2005), proteasomes themselves probably play little role in starvation-induced degradation of cellular proteins. The fact that proteasomes are restricted to degrading proteins one at a time, coupled with the high energy requirements of the ubiquitylation machinery and the proteasome itself (Peth et al., 2013b; Collins and Goldberg, 2017), likely make bulk autophagic degradation of whole proteasomes and other cellular material a more effective strategy for rapid nutrient re-mobilization than up-regulation of the UPS.

A significant barrier to the recruitment of proteasomes to phagophores is the fact that most proteasomes are located in the nucleus (Reits et al., 1997; Enenkel et al., 1998; Russell et al., 1999; Brooks et al., 2000; Pack et al., 2014; Marshall et al., 2015), whereas the autophagy machinery is found exclusively in the cytosol. Little is currently known about autophagic degradation of nuclear components. In mammals, autophagy of nuclear lamina has been reported (Dou et al., 2015), while in budding yeast, a pathway called piecemeal microautophagy of the nucleus (PMN) has been described that requires nuclear-vacuole junctions formed by Nvj1, Lam5 and Lam6 (Roberts et al., 2003; Krick et al., 2008; Mijaljica et al., 2012; Elbaz-Alon et al., 2015). More recently, selective autophagy of nuclear components mediated by the receptor Atg39 was also reported (Mochida et al., 2015), a process distinct from PMN.

Initial studies on the degradation of nuclear proteasomes following nitrogen starvation in yeast surprisingly revealed that neither Atg39-mediated nucleophagy nor components of the PMN pathway were required (Marshall et al., 2016; Waite et al., 2016; Nemec et al., 2017). Instead, a role for direct nuclear export of proteasomes mediated by the exportin Crm1 appears crucial (Stade et al., 1997; Hutten and Kehlenbach, 2007). Notably, a temperature-sensitive *CRM1* allele (termed *xpo1-1*) that strongly interferes with Crm1-dependent nuclear export substantially attenuates proteaphagy at non-permissive temperatures, although bulk autophagic flux remains unaffected (Figure 5A; Nemec et al., 2017).

In addition to Crm1, targeted deletion of a suite of autophagy components revealed many factors required for starvation-induced proteaphagy in yeast (Figure 5A). These include all subunits of the Atg1 and PI3K complexes, the entire Atg8 lipidation pathway, the Atg9 membrane delivery system, the vacuolar protease Pep4, the vacuolar phospholipase Atg15 that degrades the autophagic body membrane, and the sorting nexins Snx4/Atg24 and Snx41 or Snx42 (which function as Snx4-Snx41 or Snx4-Snx42 heterodimers, with Snx41 and Snx42 acting redundantly; Marshall et al., 2016; Waite et al., 2016; Nemec et al., 2017). Presumably, the involvement of Atg1 allows starvation signals emanating from up-stream nutrient-responsive kinases, such as Snf1 and Tor1/2 to up-regulate 26S proteasome clearance. The involvement of the sorting nexins suggests that starvation-induced proteaphagy is selective, as Snx4, Snx41 and/or Snx42 are not required for bulk autophagy in yeast (Nice et al., 2002; Reggiori and Klionsky, 2013). This mirrors the situation for ribosomes, which are selectively targeted for degradation in response to starvation (Kraft et al., 2008; Wyant et al., 2018). Snx4 is also required for autophagic clearance of the fatty acid synthase complex (Shpilka et al., 2015) and the small and large subunits of the ribosome (Nemec et al., 2017), suggesting that it might assist in degrading large protein complexes more generally. Interestingly, the sorting nexins appear to promote the formation of proteasome-containing cytoplasmic puncta that accumulate when autophagy is impaired (Waite et al., 2016; Nemec et al., 2017).

Multiple lines of evidence suggest that the CP and RP likely dissociate in the nucleus prior to their autophagic degradation. For example, turnover of the CP, but not the RP, upon nitrogen starvation in yeast was shown to be dependent upon the DUB Ubp3, suggesting that the two sub-complexes are degraded by separate routes (Waite et al., 2016; Marshall and Vierstra, 2018b). Using the “anchor-away” technique to tether the CP or RP sub-complexes in either the cytoplasm or nucleus (Haruki et al., 2008), Nemec et al. (2017) showed that disassembly of the CP, RP lid and RP base occurs prior to nuclear export, as CP or RP base degradation was not impacted when the RP lid was anchored inside the nucleus. It is currently unclear why CP-RP dissociation is necessary for proteaphagy, as fully assembled proteasomes have been reported to pass intact through the nuclear pore on their way into the nucleus (Savulescu et al., 2011; Pack et al., 2014). However, because CP activity is much lower when separated from the RP (Groll et al., 2000; Dambacher et al., 2016), dissociation of the nuclear 26S particles into sub-complexes might help attenuate CP activity until encapsulated by autophagosomes, thus preventing a sudden influx of active 26S proteasomes into the cytosol that could interfere with proteostasis in this compartment.

While proteasomes are rapidly degraded by autophagy upon nitrogen starvation, they surprisingly remain stable upon carbon starvation in both plants and yeast (Figure 5A; Waite et al., 2016; Marshall and Vierstra, 2018b), even though this treatment also activates bulk autophagy (Takeshige et al., 1992; Thompson et al., 2005; Adachi et al., 2017). Instead, carbon starvation leads to dissociation of the CP and RP, followed by their rapid export out of the nucleus and subsequent re-location into discrete PSGs that appear within an hour of transfer to carbon-free media (Figures 4B–D, 5A; Laporte et al., 2008; Marshall and Vierstra, 2018b). Surprisingly, 26S proteasome levels also remain stable upon simultaneous nitrogen and carbon starvation, implying that carbon starvation overrides the proteaphagic response elicited by the lack of nitrogen.

Cytologically, PSGs appear as membrane-less condensates that coalesce in response to the reduced ATP levels and/or cytoplasmic acidification that occur in the absence of a carbon source (Laporte et al., 2008; Peters et al., 2013; Sagot and Laporte, 2019). These puncta seemingly dissolve within minutes when carbon availability improves, suggesting that they represent a storage form of the complex. More than 40 factors have been identified that affect PSG formation (Gu et al., 2017), including Blm10 (for the CP), Spg5 and the C terminus of Rpn11 (for the RP), and the NatB N-terminal acetylation complex for both (Hanna et al., 2012; Saunier et al., 2013; Weberruss et al., 2013; van Deventer et al., 2015; Marshall and Vierstra, 2018b), but it remains unclear how many of these factors participate directly in PSG formation. By analogy with other liquid-liquid phase separation events (Alberti et al., 2019; Wang and Zhang, 2019), unstructured regions within proteasome subunits could contribute to this condensation (Aufderheide et al., 2015b).

The reasons for proteasome accretion into PSGs were initially enigmatic. However, observations that PSGs also form as yeast enter quiescence (Laporte et al., 2008), and that the sequestration of proteasomes into PSGs is antagonistic to proteaphagy (Marshall and Vierstra, 2018b), implied that PSGs act to store proteasomes under conditions that reduced growth due to lack of energy. In particular, attenuation of PSG assembly upon carbon starvation through mutants eliminating Blm10, Spg5, and NatB, or truncating Rpn11, strongly re-directs 26S proteasomes to autophagy, suggesting that proteaphagy is the default response to starvation, with PSGs providing a novel adaptation to save proteasomes during carbon stress (Marshall and Vierstra, 2018b).

The ability to store proteasomes in turn confers increased cell fitness to yeast. PSG formation during stationary phase, upon replicative aging, or in response to carbon starvation promotes rapid resumption of cell growth when nutrient availability improves (van Deventer et al., 2015; Marshall and Vierstra, 2018b), while blocking PSG formation instead suppresses the ability of cells to resume growth upon restoration of a carbon source (Marshall and Vierstra, 2018b). Presumably, the retained proteasomes enable more rapid initiation of cell division, given the importance of 26S proteasomes, and the UPS in general, for degrading regulators responsible for cell cycle progression (Ciechanover et al., 1984; Goebl et al., 1988).

Autophagic degradation of proteasomes in response to amino acid starvation has also been reported in mammals (Figure 5B; Cohen-Kaplan et al., 2016). Surprisingly, and in contrast to the situation in plants and yeast (Marshall et al., 2015, 2016), starvation-induced proteaphagy in HeLa cells is accompanied by increased subunit ubiquitylation on RPN1, RPN10 and RPN13 (Cohen-Kaplan et al., 2016). The attached poly-ubiquitin chains appear essential for proteaphagy, as siRNA-mediated silencing of the E1 or over-expression of a ubiquitin variant lacking the internal lysine residues necessary for chain concatenation reduced rates of proteasome degradation. This turnover requires the autophagy receptor p62/SQSTM1, which recognizes ubiquitylated cargo via its UBA domain and ATG8/LC3 via a canonical AIM (Noda et al., 2008, 2010; Cohen-Kaplan et al., 2016). It thus appears that, at least in the HeLa cell system, starvation induces significant ubiquitylation of proteasomes to promote recognition by the autophagy machinery (Figure 5B; Cohen-Kaplan et al., 2016).

More recently, the Atg16 homolog ATG16L1 was implicated in proteaphagy in the social amoeba *Dictyostelium discoideum* (Xiong et al., 2018). Unexpectedly, ATG16L1 directly binds to RPN1 and RPN2 *in vitro*, and co-localizes with these subunits in autophagosome-type puncta decorated with ATG8 *in vivo*. As *D. discoideum* undergoes a dramatic transformation from a single amoeba into a social pseudopod upon nutrient starvation, an appealing notion is that the interaction of ATG16L1 with 26S proteasomes provides a direct way to tether the particles to the enveloping autophagic membranes during starvation-induced proteaphagy (Xiong et al., 2018). Taken together, while starvation-induced proteaphagy is likely universal, the mechanism(s) and identity of the receptor(s) involved (if any) likely vary among eukaryotes (Marshall et al., 2015, 2016; Cohen-Kaplan et al., 2016; Xiong et al., 2018).

## Autophagic Degradation of Inactive 26S Proteasomes

In addition to starvation-induced proteaphagy, a second pathway has been described in plants and yeast that enables clearance of non-functional 26S proteasomes (Marshall et al., 2015, 2016; Nemec et al., 2017). This proteaphagic route occurs independently of the Atg1 kinase, and can be stimulated *in vivo* by treatment with chemical inhibitors, such as MG132 and bortezomib, by genetic mutations that impair CP or RP assembly, and even by pathogen effectors, such as HopM1 from *Pseudomonas syringae* (Figure 5C; Marshall et al., 2015, 2016; Üstün et al., 2018). In both *Arabidopsis* and yeast, proteasome inhibition leads to the accumulation of ubiquitylated species associated with the complex (Marshall et al., 2015, 2016). These species are not stalled targets awaiting turnover, but instead reflect extensive modification of the 26S proteasome itself (Book et al., 2010; Kim et al., 2013; Marshall et al., 2015, 2016). The identities of the modified subunits are not yet known, but analysis of the CP and RP sub-complexes individually suggests that RP subunits are dominant targets (R. S. Marshall and R. D. Vierstra, unpublished data). Subsequent studies revealed that these ubiquitin moieties are recognized by selective autophagy receptors, which then bridge the inhibited, ubiquitylated proteasomes to Atg8 lining the expanding phagophore (Figure 5C).

The autophagy receptor for clearing inhibited proteasomes in *Arabidopsis* is RPN10, which uses two distinct UIMs to tether proteasomes to the enveloping autophagic vesicle. One UIM binds the ubiquitin moieties attached to 26S proteasomes, while the other surprisingly binds ATG8 (Marshall et al., 2015, 2019). This non-canonical, UIM-mediated interaction between RPN10 and ATG8 is striking, as it does not involve the canonical LIR/AIM docking site (LDS) on ATG8, but instead requires an alternative hydrophobic patch recently termed the UIM docking site (UDS; Marshall et al., 2019). The yeast version of Rpn10 is truncated compared to its *Arabidopsis* counterpart, meaning it lacks the Atg8-binding UIM sequence and consequently has no discernable role in proteaphagy. Instead, Cue5 acts as the yeast receptor for ubiquitylated proteasomes, using a CUE domain to bind ubiquitin and a canonical AIM to bind Atg8 (Figure 5C; Marshall et al., 2016). The UIM1-UIM2 pairing for *Arabidopsis* RPN10 and the CUE-AIM pairing for Cue5 thus provides an elegant example of convergent evolution, where different interacting motifs are exploited to generate the same outcome, namely tethering of ubiquitylated proteasomes to autophagic membranes.

Cue5 and its human counterpart TOLLIP have been implicated in the autophagic clearance of various aggregation-prone proteins (Lu et al., 2014) and, intriguingly, inhibitor-induced proteaphagy in yeast is likewise preceded by aggregation of 26S proteasomes into peri-vacuolar insoluble protein deposit (IPOD)-type structures (Kaganovich et al., 2008; Marshall et al., 2016), suggesting some degree of overlap between the proteaphagy and aggrephagy machineries. The IPODs seen upon proteasome inhibition are distinct from PSGs (Marshall and Vierstra, 2018b), although there might be some overlap between the two types of puncta during early stages of carbon starvation (Peters et al., 2016). The two condensates can be easily distinguished based on their co-localization with either Blm10 (in PSGs) or the aggregation-prone prion protein Rnq1 (in IPODs; Figure 4B).

IPOD formation is dependent on the oligomeric chaperone Hsp42, which helps coalesce aggregated proteins (Specht et al., 2011; Malinovska et al., 2012; Miller et al., 2015). The accumulation of yeast 26S proteasomes into IPODs upon inhibition, and their subsequent autophagic breakdown, were also found to require this aggregase (Marshall et al., 2016). Where inactive 26S proteasomes become ubiquitylated is currently unclear; one possibility is that dysfunctional proteasomes are first ubiquitylated and then delivered to IPODs with the help of Hsp42, while the other is that Hsp42 first delivers dysfunctional proteasomes into IPODs, which are then ubiquitylated through one or more IPOD-resident E3s.

Whereas chemical inhibitors compromising the CP induce autophagic degradation of both CP and RP, possibly due to the tighter interaction between the two sub-complexes that allosterically results from CP inhibition (Kleijnen et al., 2007), mutations that compromise proteasome assembly instead appear to induce turnover of the affected CP and RP sub-particles separately. For example, the *doa5-1* allele that compromises the α_5_ subunit of the CP triggers the Cue5-dependent turnover of the rest of the CP, but not the RP, while the *rpn5*Δ*C* mutation impacting Rpn5 triggers the Cue5-dependent turnover of the rest of the RP, but not the CP (Marshall et al., 2016). These observations imply that proteaphagy can be initiated for both the whole 26S particle, and for the individual CP and RP sub-complexes separately.

Clearly, an important feature of inhibitor-induced proteaphagy is its ability to discriminate between functional and dysfunctional particles. One possibility is that stalled or compromised 26S proteasomes acquire a distinct conformation that is recognized by Hsp42 and/or the ubiquitylation machinery, which directs their accumulation into IPODs. An intriguing factor in this scenario was Ecm29, as it binds specifically to mutant forms of 26S proteasomes, and thus could detect inappropriate conformations induced by inactivation (Lehmann et al., 2010; Lee S. Y. et al., 2011; Panasenko and Collart, 2011; Park et al., 2011). However, analysis of yeast Δ*ecm29* mutants suggested this it is not involved in proteaphagy (Marshall and Vierstra, 2018b). Several E3s have been detected in association with 26S proteasomes that could instead provide this quality control (Xie and Varshavsky, 2000; Crosas et al., 2006; Panasenko and Collart, 2011), some of which ubiquitylate specific subunits (Besche et al., 2014; Fu et al., 2018), but their function(s) in relation to proteaphagy, if any, remain to be determined. Further work is certainly required to fully unravel the mysteries surrounding this last chapter in the life of a proteasome.

## Conclusions and Perspectives

Since the discovery of the UPS over three decades ago, great progress has been made in our understanding of selective proteolysis by this system. This includes intricate knowledge of the 26S proteasome itself, which combines strict substrate selectivity with extreme promiscuity with respect to substrate processing to enable the degradation of thousands of proteins with exquisite specificity. Recent technological advances in cryo-EM imaging have generated increasingly detailed models describing substrate recognition and processing by the 26S proteasome (Lander et al., 2012; Lasker et al., 2012; Bhattacharyya et al., 2014; de la Peña et al., 2018; Dong et al., 2019; Finley and Prado, 2019). In parallel, a multitude of additional studies across several kingdoms have advanced our knowledge of the 26S proteasome life cycle, including its biosynthesis, assembly, localization, and ultimately turnover (Figure 6; Collins and Goldberg, 2017; Rousseau and Bertolotti, 2018). The combined studies reveal the use of common mechanisms to control 26S proteasome assembly, activity, and degradation, though often by exploiting distinct factors and machineries.

However, despite these advances, much remains unknown. Areas of continued uncertainty include, but are not limited to: (i) which transcription factors are responsible for proteasome gene expression under non-stressed conditions in plants and yeast; (ii) the identities of additional proteasome assembly chaperones, particularly for the RP lid; (iii) how ubiquitin-chain topologies and the geometric or structural features of the substrate influence recognition and turnover by the proteasome; (iv) how extrinsic factors, proteasome-interacting proteins, and post-translational modifications regulate the various proteasome activities; (v) whether proteasomes are selectively chosen for proteaphagy during nutrient starvation using dedicated receptor(s), or degraded in bulk along with the rest of the cytoplasm; and (vi) how dysfunctional proteasomes are detected prior to autophagic degradation, and which subunit(s) are ubiquitylated by which E3(s).

The UPS is involved in nearly all cellular processes in eukaryotes, and its mis-regulation often contributes to aging and disease, or loss of crop yield (Saez and Vilchez, 2014; Rape, 2018; Li et al., 2019). This has fed a desire to understand the dynamic regulation of proteasomes, simultaneously advancing our knowledge of basic cellular processes that control this proteolytic machine, and providing a potential avenue for the development of novel therapies to ameliorate a variety of diseases related to 26S proteasomes and their activity.

## Author Contributions

RSM and RDV conceived the article, prepared the figures, and wrote the manuscript. Both authors have made a substantial, direct and intellectual contribution to the work, and approved it for publication.

### Conflict of Interest Statement

The authors declare that the research was conducted in the absence of any commercial or financial relationships that could be construed as a potential conflict of interest.
